# A Si‐MoSe_2_ Heterostructured Anode with Enhanced Thermal Transport and Electrochemical Performance for Liquid and All‐Solid‐State Lithium‐Ion Batteries

**DOI:** 10.1002/advs.202523320

**Published:** 2026-01-31

**Authors:** Yajun Zhu, Jiaqi Gu, Guangwu Zhang, Tianli Han, Yang Lu, Zhongbing Li, Hang Su, Fei Wang, Haojun Xu, Wentuan Bi, Qiye Zheng, Jinyun Liu

**Affiliations:** ^1^ Key Laboratory of Functional Molecular Solids Ministry of Education College of Chemistry and Materials Science Anhui Normal University Wuhu Anhui P. R. China; ^2^ Institute of Energy Hefei Comprehensive National Science Center Hefei Anhui P. R. China; ^3^ Anhui Deeiot Energy Technology Co., Ltd Wuhu Anhui P. R. China; ^4^ Department of Mechanical and Aerospace Engineering The Hong Kong University of Science and Technology Hong Kong SAR P. R. China

**Keywords:** cycling stability, heterostructure, Li‐ion battery, silicon anode, thermal transport

## Abstract

Silicon anodes offer high theoretical capacity for lithium‐ion batteries but suffer from volume‐change‐induced instability and degradation. Conventional van der Waals coatings yield unstable interfaces and poor ion/electron transport, while thermal transport remains underexplored. Here, we propose heterointerface‐engineered Si@MoSe_2_@C anodes with chemically bonded interfaces, where lattice‐matched MoSe_2_ covalently bridges porous Si and carbon coating, forming robust Si─Se─Mo linkages that stabilize the structure and optimize transport pathways. The Si@MoSe_2_@C anode delivers 1054 mAh g^−1^ after 100 cycles at 0.2 A g^−1^—exceeding most Si anodes—and 99.5% Coulombic efficiency over 400 cycles at 1.0 A g^−1^, with high cycling efficiency demonstrated in both liquid and all‐solid‐state lithium‐ion batteries (ASSLIBs). In situ X‐ray diffraction, Raman spectroscopy, and electron microscopy/spectroscopy, together with first‐principles calculations, confirm that this MoSe_2_‐mediated covalent bridging enables reversible reactions with favorable kinetics and structural integrity by strengthening and delocalizing Se─Si bonding and reducing Li^+^ migration barriers by 24%. Critically, we present the first measurements of the effective thermal conductivity of a silicon‐anode composite, showing that Si@MoSe_2_@C exhibits a 27% higher value than Si, addressing long‐overlooked cell‐level thermal‐management requirements and improving elevated‐temperature cell performance. This heterointerface design provides a synergistic strategy for engineering high‐performance Si anodes across batteries with enhanced safety.

## Graphical abstract

Chemically bonded Si@MoSe_2_@C heterointerfaces with robust Si─Se─Mo bonds enable high‐performance Si anodes. Lattice‐matched MoSe_2_ on porous Si with carbon‐protective coating delivers 1054 mAh g^−1^ after 100 cycles and 99.5% Coulombic efficiency over 400 cycles. Reduced interfacial resistance enhances kinetics and yields 27% higher thermal conductivity, providing superior cycling stability and improved safety in liquid and all‐solid‐state cells.

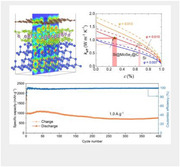

## 1 Introduction

The global shift to renewable energy and electrified transport is accelerating the demand for advanced energy storage, positioning high‐performance lithium‐ion batteries as a central focus of research for a sustainable, low‐carbon future [1, 2]. Among various anode candidates, silicon (Si) stands out for its exceptionally high theoretical specific capacity of ∼4200 mAh g^−1^, which is over ten times higher than that of graphite anode 372 mAh g^−1^ [3, 4]. However, the practical application of Si anode is severely limited by its drastic volume changes, exceeding 300% during lithiation and delithiation. Such volumetric fluctuations can induce mechanical degradation and pulverization, detachment of active materials from current collectors, and continuous formation of unstable solid–electrolyte interphase (SEI) layers, collectively resulting in rapid capacity fading and short cycle life [5, 6].

To address these instability challenges of Si anodes, a variety of strategies have been developed [7, 8, 9]. Common approaches include nanostructuring Si composites to accommodate strains [10]. For example, some achievements used few‐layer MoS_2_ nanosheets to wrap Si nanoparticles to construct a heterostructure [11], and prepared porous or core–shell architectures with elastic binders to buffer expansion [12]. Surface coatings [13], particularly carbon layers, have been widely explored to buffer mechanical stress and promote stable SEI formation. However, conventional coating methods often lack the mechanical robustness to withstand the extreme volume expansion of Si, leading to cracking, loss of protection, and interfacial delamination due to weak van der Waals interactions between carbon and Si [14]. Despite advances in coating methods, such as yolk–shell and hollow structures that introduce voids between the Si core and carbon shell to better accommodate volume changes, persistent challenges remain [10]. Issues such as lattice mismatch, material incompatibility, and unfavorable energy level alignment can hinder interfacial stability [15], while insufficient interfacial strength and suboptimal ion, electron, and heat transport between Si and the coating further limit performance [16]. Thus, constructing robust and well‐matched heterogeneous interface architectures between Si and the coating layer remains a critical yet challenging goal for improving the stability and overall performance of Si‐based electrodes.

Recently, transition metal dichalcogenides (TMDs) have emerged as a powerful platform for designing heterogeneous interfaces across various fields, thanks to their layered structures and unique chemical properties [17]. In TMDs, atomic layers with covalent (X─M─X) linkages are bonded by weak van der Waals forces, which not only facilitate efficient lithium‐ion (Li^+^) insertion and extraction [18], but also enable tunable interfacial engineering. Among TMDs, MoSe_2_ stands out due to its large interlayer spacing (∼6.4 Å) and high electrical conductivity (>100 S cm^−1^ for nanofilms), which lowers the energy barrier for electron transfer and reduces overall resistance when used in composites [19]. These properties make MoSe_2_ a promising building block for interface engineering in battery electrodes. Prior MoSe_2_‐on‐Si studies showed that edge‐rich or chemically anchored MoSe_2_ layers engaged interfacial processes at moderate potentials, regulated early interphase formation, and stabilized cycling—supporting MoSe_2_’s role as an electronically competent interfacial modifier rather than a bulk capacity host [20]. In that work, a pronounced high‐potential reduction feature near ∼1.5 V vs Li/Li^+^ signaled early interfacial engagement; such front‐loaded chemistry is advantageous, as it templates a uniform, conductive SEI and reduces polarization before Si is driven toward ∼0 V—illustrating that a modestly higher reaction potential for a thin MoSe_2_ interlayer is an efficient feature. While beyond Li‐ion, MoSe_2_ working with carbon as anode for potassium‐ion batteries [21], also indicated that confining MoSe_2_ within a composite could stabilize the interface and reduce electrochemical polarization.

In addition, while the electrochemical performance of Li‐ion battery (LIB) electrodes has been extensively investigated, their thermal transport properties have received far less attention [20], despite being critically important for safe and reliable battery operation. This gap is especially notable for Si electrodes. On the one hand, the effective thermal conductivity (*k*) of electrode materials, influenced by the battery fabrication and degradation, is strongly correlated to the electrical transport properties [22, 23], which can serve as an indicator of the battery health status [24, 25]. On the other hand, considering the high *k* of the current collectors, the effective *k* of the electrode materials in battery cells largely dictates heat dissipation during charging/discharging [26]. Recent studies have highlighted that poor thermal transport within electrode layers can lead to localized overheating, which may accelerate capacity fading or even trigger thermal runaway [27, 28, 29, 30]. Therefore, a direct characterization of 𝑘 for Si anode composites provides valuable insight into the structure‐property‐function relationships and improving *k* is essential for ensuring cycling stability and safety [31, 32].

Here, we propose MoSe_2_ as a coating material for Si anodes to achieve high interfacial stability through the construction of a heterogeneous Si@MoSe_2_ interface to enhance the electrochemical, mechanical, and thermal performance of the Si anode. At the chemical level, the coupling between MoSe_2_ and Si can be regulated to form strong covalent interfacial bonding with a strength significantly higher than that of traditional coatings [33]. Meanwhile, the high lattice compatibility between the (002) plane of MoSe_2_ and the (111) plane of Si enables the formation of a robust epitaxial heterostructure that facilitates the coating stability [20].

Building on these insights, we develop a heterostructured Si‐based anode consisting of MoSe_2_@C coating on porous Si (denoted as Si@MoSe_2_@C). Prior Si‐anode strategies often relied on high‐temperature carbothermal treatments (∼800°C) that primarily provide physical confinement and improved percolation rather than robust chemical bonding [34]. As an example, a Si@MoSe_2_ core–shell was prepared via amino modification and selenization, where the MoSe_2_ layer mainly regulated SEI formation through physical isolation [20]. However, achieving strong, intrinsic chemical bonding at the Si–coating interface remains challenging. In our study, unsaturated Si─O bonds are introduced on the surface of Si particles through magnesiothermic reduction, resulting in oxygen vacancies that facilitate the formation of strong Si─Se─Mo bonds upon hydrothermal deposition of MoSe_2_, creating a robust Si@MoSe_2_ heterointerface. This engineered interface provides efficient charge transport pathways for electrochemical reactions and enhances the protective effect of MoSe_2_ on the Si core. To further stabilize the structure, a carbon layer is coated onto the Si@MoSe_2_, effectively immobilizing the MoSe_2_ and improving overall electrode stability.

We employ in situ X‐ray diffraction (XRD) and Raman spectroscopy along with transmission electron microscopy (TEM), X‐ray photoelectron spectroscopy (XPS), electron paramagnetic resonance (EPR), and a series of systematic electrochemical characterization to demonstrate the chemical and structural advantage of the Si@MoSe_2_@C as well as the reversible phase transitions and bonding evolution during lithiation and delithiation processes. Critically, MoSe_2_ here is not intended to contribute significant capacity; it is engineered as an ultrathin, electronically competent, ion‐permeable interlayer. Density‐functional theory (DFT) calculations—via projected crystal orbital Hamilton population (pCOHP) and electron localization function (ELF) analyses—reveal strong Si─Se─Mo interfacial bonding and interfacial charge localization/bridging, which i) strengthen adhesion and mechanical integrity, ii) lower the Li^+^ migration barrier across the interface, and iii) enhance interfacial electronic transport. Consistent with these predictions, the Si@MoSe_2_@C anode exhibits high specific capacity with excellent rate performance and slow capacity fade, reflecting reduced kinetic polarization and a stabilized, thin SEI while keeping the working capacity Si‐dominated at low potentials. Although thermal transport is critical for fast charging and safety, it is rarely quantified for anodes; to our knowledge, this is the first direct report of thermal conductivity for a silicon‐anode composite, together with a mechanistic interpretation that links interface chemistry to electrode‐scale heat flow.

## 2 Results and Discussion

### 2.1 Synthesis and Structural Characterization of Si@MoSe_2_@C Heterostructures

**FIGURE 1:**
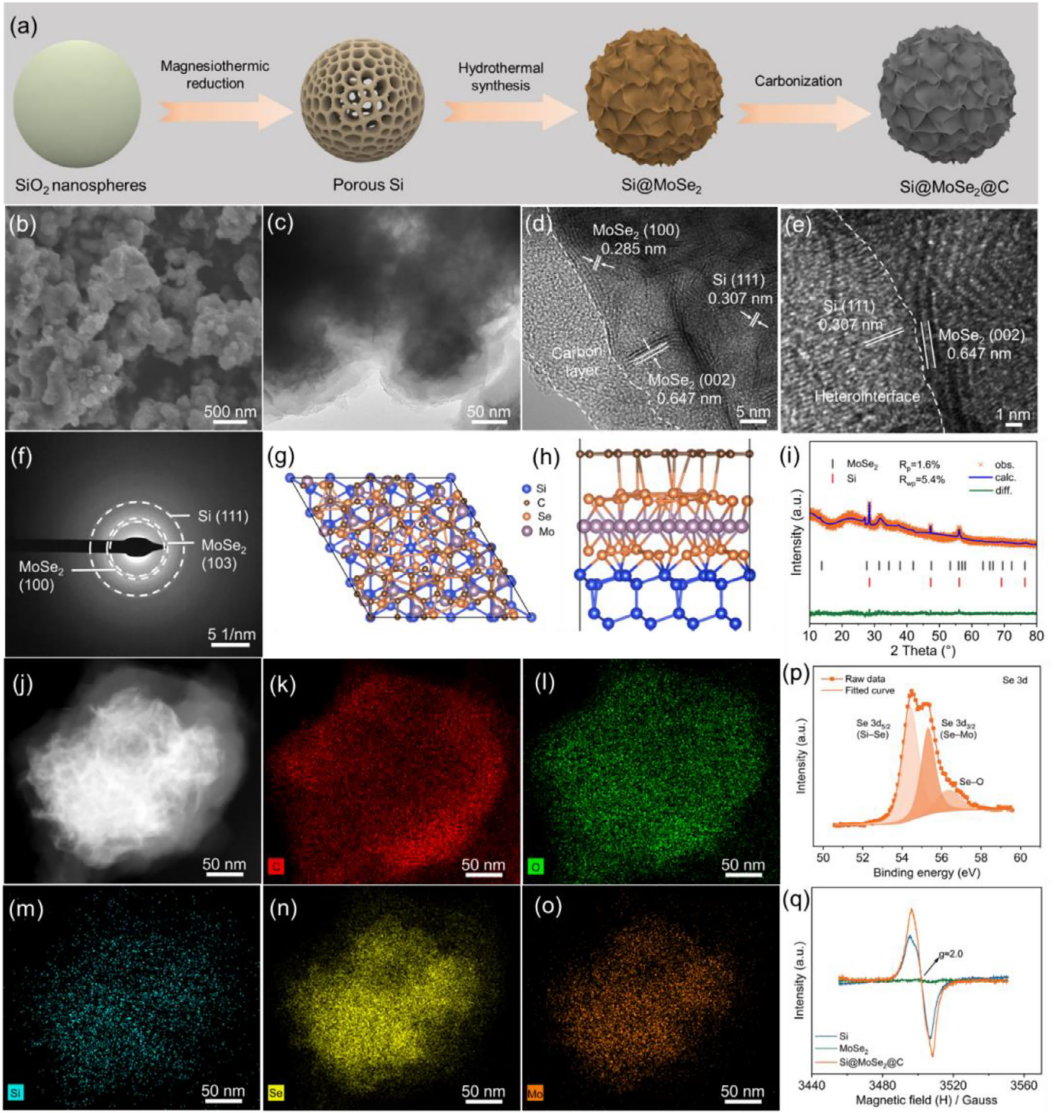
(a) Schematic illustration of the synthesis process, (b) SEM, (c) TEM, (d, e) HRTEM images of the heterostructured Si@MoSe_2_@C, and (f) the corresponding HRTEM‐SAED pattern. (g, h) Top‐ and side‐view of the atomic models of the Si@MoSe_2_@C heterostructure based on the HRTEM and XPS data for later DFT calculation. (i) XRD Rietveld refinement pattern, (j) SEM image, and elemental mappings of (k) C, (l) O, (m) Si, (n) Se, and (o) Mo. (p) High‐resolution XPS spectrum of Se 3d in Si@MoSe_2_@C. (q) EPR spectra of Si, MoSe_2_, and Si@MoSe_2_@C.

Figure 1a illustrates the three‐step synthesis strategy for preparing Si@MoSe_2_@C composites. The process begins with magnesiothermic reduction of commercially available SiO_2_ nanospheres to generate porous Si with abundant oxygen vacancies (Figure S1a). Subsequently, MoSe_2_ is hydrothermally deposited onto the Si surface, forming robust Si─Se─Mo interfacial bonds (Figure S1b). For electrochemical benchmarking, the porous Si obtained in the first step and MoSe_2_ synthesized under identical conditions but without Si were tested as control electrodes. The synthesis concludes with carbon coating to yield the final Si@MoSe_2_@C architecture (Figure 1b). Thermogravimetric analysis (TGA, in the air, Figure S2) shows a net mass loss between ∼330°C–500°C that we attribute to i) oxidative volatilization of carbon to CO_2_ and ii) oxidative decomposition of MoSe_2_ with formation and sublimation of SeO_2_ [35]. The combined mass loss assigned to CO_2_ and SeO_2_ is 47.4 wt.%. Consistent with prior TGA‐air studies on carbons coated Si anodes and NMC cathode, the 400°C–500°C window is dominated by carbon combustion to CO_2_ [36, 37], from which we determine a carbon content of 15.6 wt.% in the Si@MoSe_2_@C composite. The remaining loss within 330°C–500°C is assigned to SeO_2_ volatilization from MoSe_2_ oxidation. To further constrain the composite composition beyond TGA‐based deconvolution, an independent, element‐specific analysis was performed using inductively coupled plasma–optical emission spectroscopy (ICP–OES). The Mo content measured by ICP–OES was converted to the corresponding MoSe_2_ mass fraction (≈48 wt.%) using the molar‐mass ratio of MoSe_2_ to Mo, assuming that Mo in the as‐prepared composite is predominantly present as MoSe_2_, as supported by XRD phase identification and Mo─Se chemical‐state analysis from XPS. The carbon content (15.6 wt.%) was taken from the 400°C–500°C region of the TGA curve, where carbon combustion dominates, and the remaining mass fraction was assigned to Si.

TEM analysis confirms the successful formation of a well‐defined core–shell Si@MoSe_2_@C composite particle with a hierarchically porous structure (Figure 1c). The Brunauer–Emmett–Teller (BET) surface area measurements quantify the porosity evolution throughout synthesis. As can be seen from Figure S3, pristine porous Si exhibits a specific surface area of 157.15 m^2^ g^−1^, which decreases to 72.90 m^2^ g^−1^ upon hydrothermal MoSe_2_ deposition in Si@MoSe_2_ (the value for pure MoSe_2_ is 24.31 m^2^ g^−1^). The final Si@MoSe_2_@C composite exhibits a surface area of 22.44 m^2^ g^−1^ after carbon coating. This controlled reduction in surface area is advantageous for suppressing undesirable side reactions with the electrolyte during electrochemical cycling.

High‐resolution TEM (HRTEM) imaging reveals distinct crystalline lattice fringes with d‐spacings of 0.307, 0.285, and 0.647 nm, corresponding to the (111) plane of Si, and the (100) and (002) planes of MoSe_2_, respectively (Figure 1d,e). The outer carbon shell exhibits a uniform thickness of approximately 7–10 nm, providing effective encapsulation of the underlying heterostructure. Selected area electron diffraction (SAED) analysis confirms the polycrystalline nature of the constituent Si and MoSe_2_ phases (Figure 1f). Experimentally‐informed atomic model of the Si@MoSe_2_ interface refined by geometric optimization is presented in Figure 1g,h, serving as the structural basis for DFT calculation; and the Si@C structure is displayed in Figure S4. XRD with Rietveld refinement unambiguously identifies the crystalline phases of each component (Figure 1i), with reference comparisons for pure Si and MoSe_2_ phases provided in Figures S5 and S6. Energy‐dispersive X‐ray spectroscopy (EDS) elemental mapping demonstrates uniform spatial distribution of carbon, O, Si, Se, and Mo throughout individual Si@MoSe_2_@C particles (Figure 1j–o).

Comprehensive chemical state analysis was conducted using high‐resolution XPS and EPR to elucidate the composition and bonding characteristics of the as‐synthesized Si@MoSe_2_@C composite. We note that XPS does not provide a distinct “Si─Se─Mo” ternary‐bond peak; instead, interfacial chemical coupling is inferred from consistent chemical‐state signatures across Se 3d, Si 2p, and Mo 3d (corroborated by FTIR in Figure S9). The Si 2p spectrum reveals Si─Si (Si 2p_3/2_) bond at 98.9 eV and Si─Si (Si 2p_5/2_) band at 99.7 eV, alongside Si─O bonds at 103.2 eV, confirming a surface oxide layer (Figure S7a,b) [38]. Carbon states are evidenced by C 1s peaks at 284.3 and 285.6 eV, corresponding to C─C and C─O bonds, respectively (Figure S7c). The Mo 3d region exhibits peaks at 228.4, 229.9, and 231.6 eV, attributed to Mo 3d_5/2_, Mo^4+^, and Mo 3d_3/2_ states, respectively (Figure S7d). In the Se 3d spectrum (Figure 1p), doublet‐constrained fitting resolves a selenide component assigned to Se─Mo (MoSe_2_; Se 3d_3/2_ at ∼55.3 eV, within the reported MoSe_2_ range) together with a minor oxidized Se─O contribution (∼56.4 eV); importantly, an additional low‐binding‐energy component at ∼54.5 eV is assigned to interfacial Si─Se (Se 3d_5/2_), supporting chemical coupling at the Si/MoSe_2_ interface rather than a purely physisorbed coating [39, 40].

The O 1s spectrum further corroborates the interface chemistry, with peaks at 532.6 and 533.5 eV corresponding to Si─O and C─O bonds, respectively (Figure S7e) [41]. EPR spectroscopy in Figure 1q reveals a clear signal at g ≈2.0 for the magnesiothermically reduced porous Si and Si@MoSe_2_@C (retained after MoSe_2_ growth/carbon coating), consistent with oxygen‐deficiency‐related paramagnetic centers generated during reduction [42]. Taken together, these XPS/EPR (and FTIR) results support chemically coupled Si─Se/Se─Mo heterointerfaces, while oxygen‐deficient surface SiO_x_ can modulate electronic configurations and amplify localized electric‐field effects [43].

Based on these chemical state characterizations, the formation of the heterogeneous Si@MoSe_2_ interface can be attributed to coordinated redox and bonding reactions during hydrothermal synthesis. The XPS‐confirmed presence of Mo^4+^ states and Si─Se bonds suggests that O^2−^ ions in MoO_4_^2−^ precursors act as reducing agents, driving the valence state transition of Mo^6+^ to Mo^4+^. This process enables the epitaxial growth of MoSe_2_ heterostructures directly on the Si surface, with simultaneous generation of Si─Se interfacial bonds as evidenced by the characteristic XPS peak at 55.0 eV (Figure 1p).

The structural composition and bonding characteristics of the as‐prepared Si@MoSe_2_@C composite were investigated using complementary vibrational spectroscopic techniques. (Figure S8a). The Raman spectrum demonstrates characteristic D and G bands of the carbon coating at 1367 and 1578 cm^−1^, respectively. Deconvolution analysis reveals four distinct peaks (Figure S8b), where the broad D band at 1367 cm^−1^ contains contributions from both disorder in sp^2^‐hybridized carbon and sp^3^‐hybridized carbon (diamond‐like carbon), typical of pyrolyzed carbon coatings, while the G band at 1578 cm^−1^ corresponds to in‐plane stretching vibrations of sp^2^‐hybridized carbon in graphitic carbons [44]. Fourier transform infrared (FTIR) spectroscopy provides complementary evidence for interfacial bonding (Figure S9). Pristine Si exhibits a characteristic peak at 473 cm^−1^, attributed to the vibration of Si─O bond [45], and a band at 1093 cm^−1^ corresponding to Si─Si vibrations. After the carbonization process, the 1093 cm^−1^ band of Si@MoSe_2_@C splits into two bands at 1230 and 1104 cm^−1^, which can be attributed to interfacial chemical bonding between Si and the MoSe_2_ layer [46].

### 2.2 Electrochemical Performance and Cycling Stability

**FIGURE 2:**
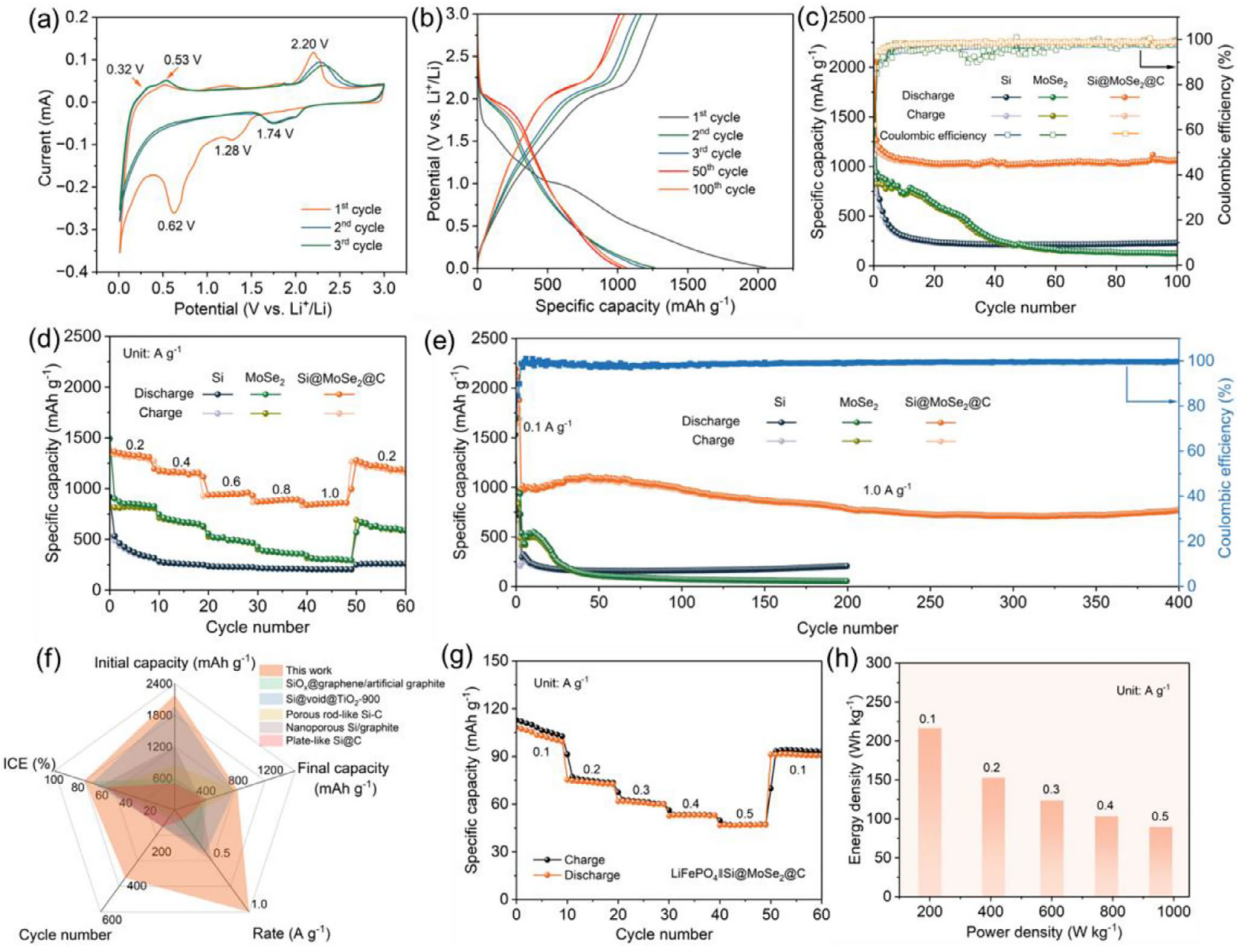
(a, b) Cyclic voltammetry (CV) profiles of the Si@MoSe_2_@C anode recorded at 0.1 mV s^−1^ (a) and the corresponding galvanostatic charge–discharge curves measured at 0.2 A g^−1^ (b). (c–e) CV profiles over 100 cycles with the corresponding Coulombic efficiency (CE) evolution (c), galvanostatic charge–discharge curves (d), and extended cycling at 1.0 A g^−1^ over 400 cycles of the Si@MoSe_2_@C anode, compared with the control Si and MoSe_2_ electrodes measured under identical conditions. (f) Radar chart providing multidimensional performance comparison with Si‐based anodes across key metrics. (g) Rate‐performance of LiFePO_4_||Si@MoSe_2_@C full‐cell. (h) Ragone plot showing energy and power density capabilities based on active material mass.

**FIGURE 3:**
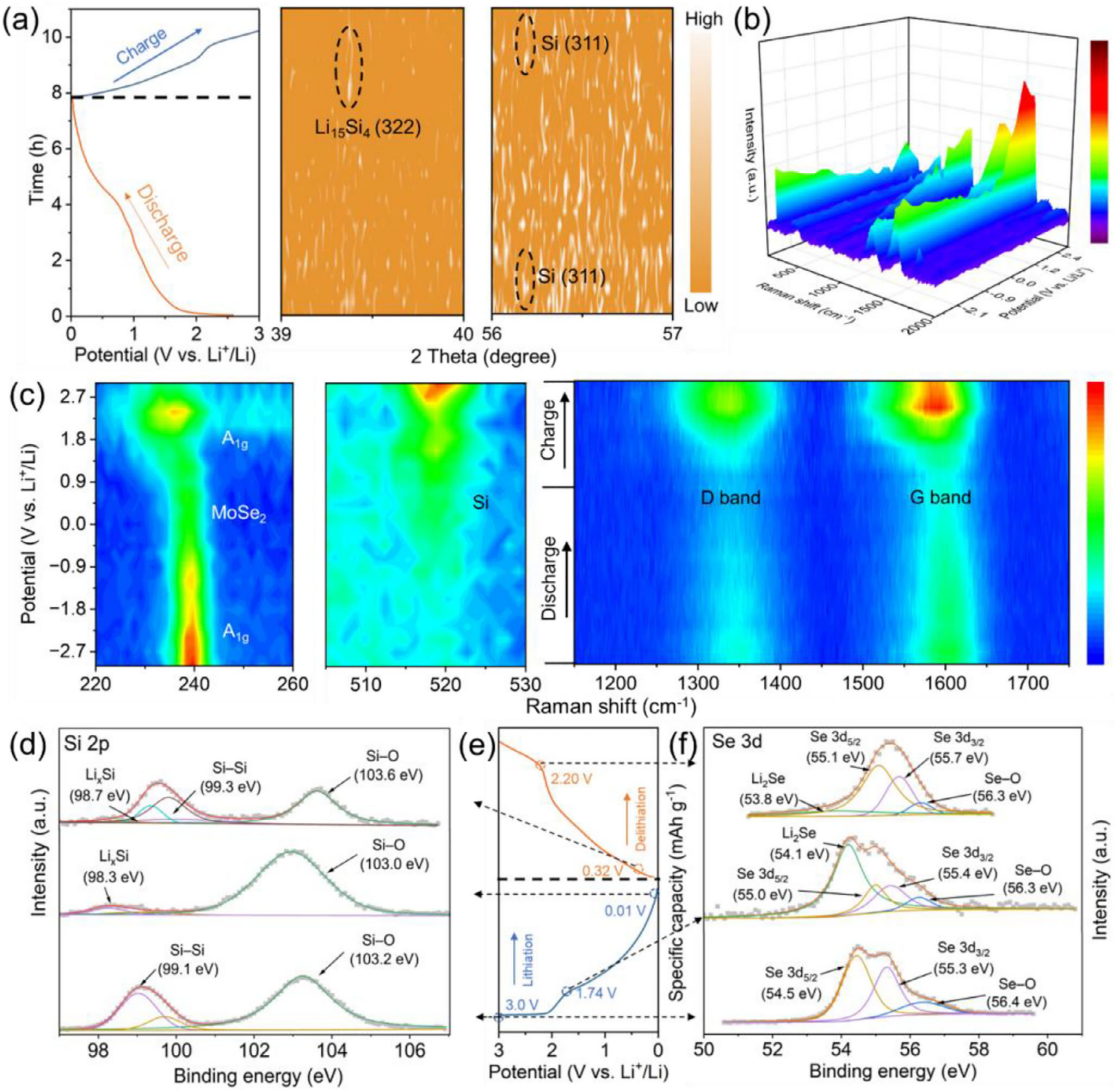
(a) Charge–discharge profiles and the corresponding in situ XRD contour plots of the Si@MoSe_2_@C anode during lithiation/delithiation. (b, c) In situ Raman spectra during one complete charge–discharge cycle. (d) Ex situ XPS analysis of Si 2p spectra at different charge states. (e) Corresponding charge–discharge curves with marked XPS measurement points. (f) Se 3d spectra evolution during lithiation and recovery during delithiation.

**FIGURE 4:**
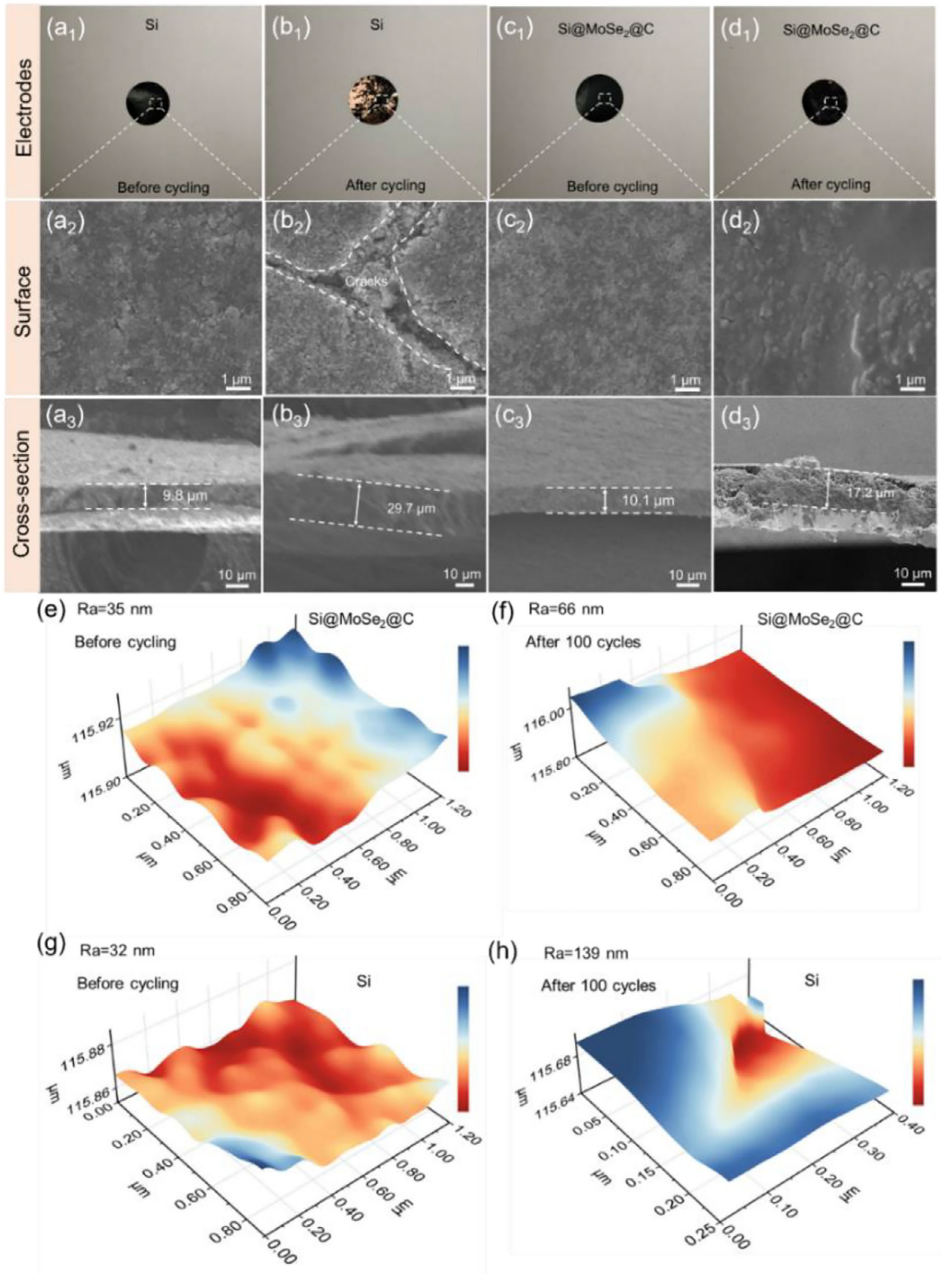
(a_1_–b_3_) Pure Si anode showing severe delamination, surface cracking, and thickness expansion after cycling. (c_1_–d_3_) Si@MoSe_2_@C anode maintaining structural integrity with suppressed thickness increase. 3D optical surface analysis revealing surface roughness changes for (e, f) Si@MoSe_2_@C compared to (g, h) Si after 100 cycles.

The electrochemical performance of the Si@MoSe_2_@C anode was evaluated and compared with individual Si and MoSe_2_ components (Figure 2). Cyclic voltammetry (CV) analysis reveals the distinct electrochemical processes occurring within the composite electrode (Figure 2a). In the first cathodic scan (Figure 2a), the reduction peak at ∼1.28 V is assigned to initial Li^+^ intercalation into the layered MoSe_2_ component (formation of Li_x_MoSe_2_), which in MoX_2_ (X = S/Se) systems occurs near ∼1.3 V and is often coupled to an early‐stage 2H → 1T/1T’‐type structural transition [18, 47]. By contrast, the broader low‐potential reduction features are attributed to overlapping conversion/SEI processes and the onset of Si alloying at much lower potentials (Si alloying mainly below ∼0.3 V; SEI/oxide‐related processes typically appear around ∼0.8–0.6 and ∼0.45 V, respectively) [18, 47]. The reduction peak around 0.15 V is attributed to the lithiation of crystalline Si to form amorphous Li_x_Si alloys, while the corresponding anodic peaks at 0.32 and 0.53 V represent the delithiation process. Additionally, the stable reduction peak near 1.74 V indicates Li^+^ intercalation into MoSe_2_ followed by conversion to metallic Mo, with the oxidation peak at 2.20 V corresponding to the reverse Mo‐to‐MoSe_2_ transformation [48]. Such electrochemical reversibility is further corroborated by in‐situ structural and spectroscopic study during cycling in Figures 3 and post‐cycling analysis in Figure 4.

**FIGURE 5:**
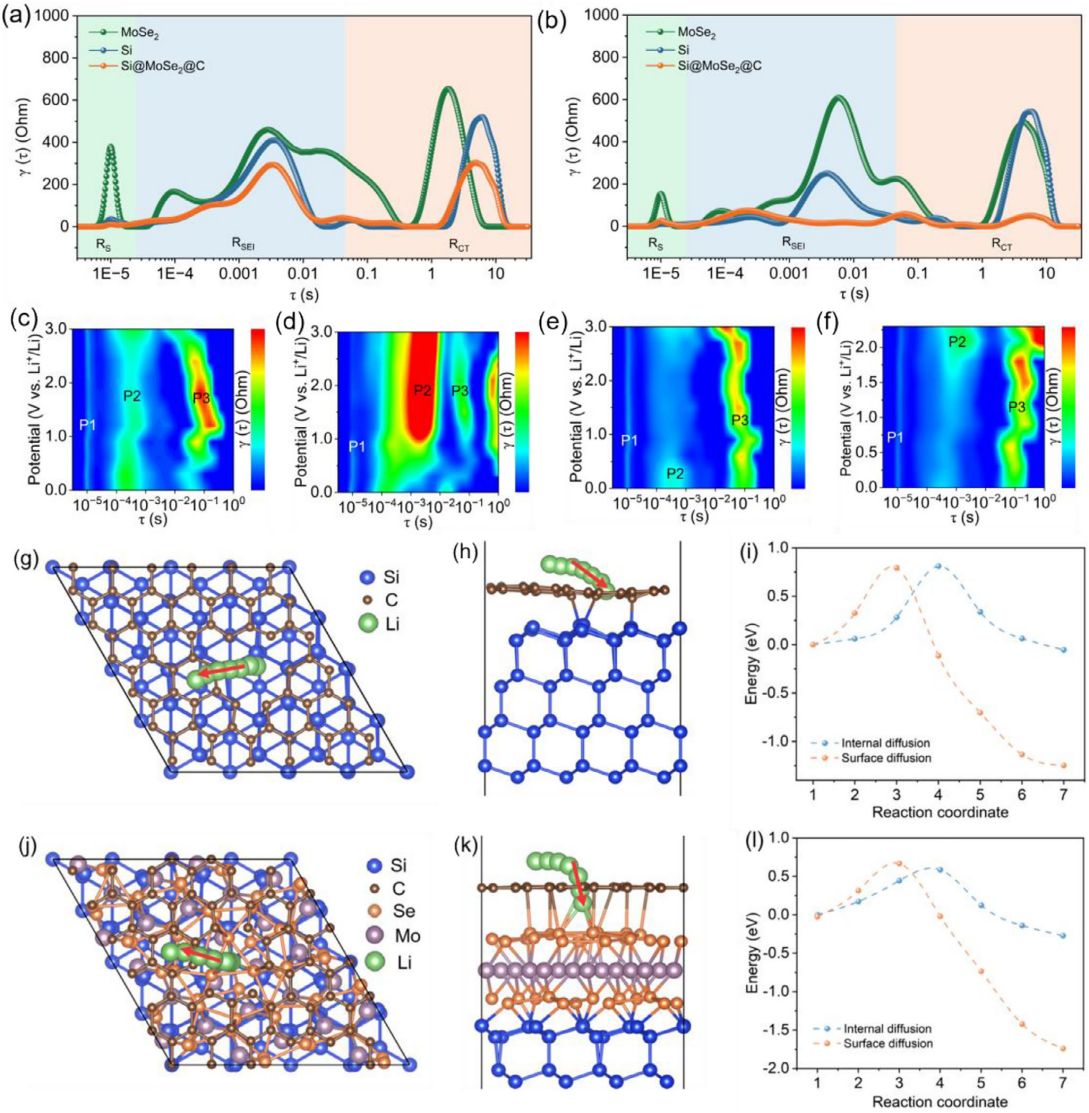
DRT curves for Si, MoSe_2_, and Si@MoS_2_@C anodes (a) before and (b) after 100 cycles. (c–f) DRT contour plots of Si@MoSe_2_@C with resistance components P1 (*R*_S_), P2 (*R*_SEI_), and P3 (*R*_CT_) during (c) initial charging, (d) initial discharging, (e) charging, and (f) discharging after 100 cycles. (g–l) Comparison of DFT‐calculated Li–ion migration pathways and energy landscapes in traditional Si@C (g–i) and Si@MoSe_2_@C heterostructure (j–l). Panels (g, j) and (h, k) show the optimized atomic structures in top and side views, respectively, with overlaid Li‐ion diffusion trajectories from DFT calculations. Panels (i, l) present the corresponding energy profiles, revealing significantly reduced migration barriers in the Si@MoSe_2_@C system.

**FIGURE 6:**
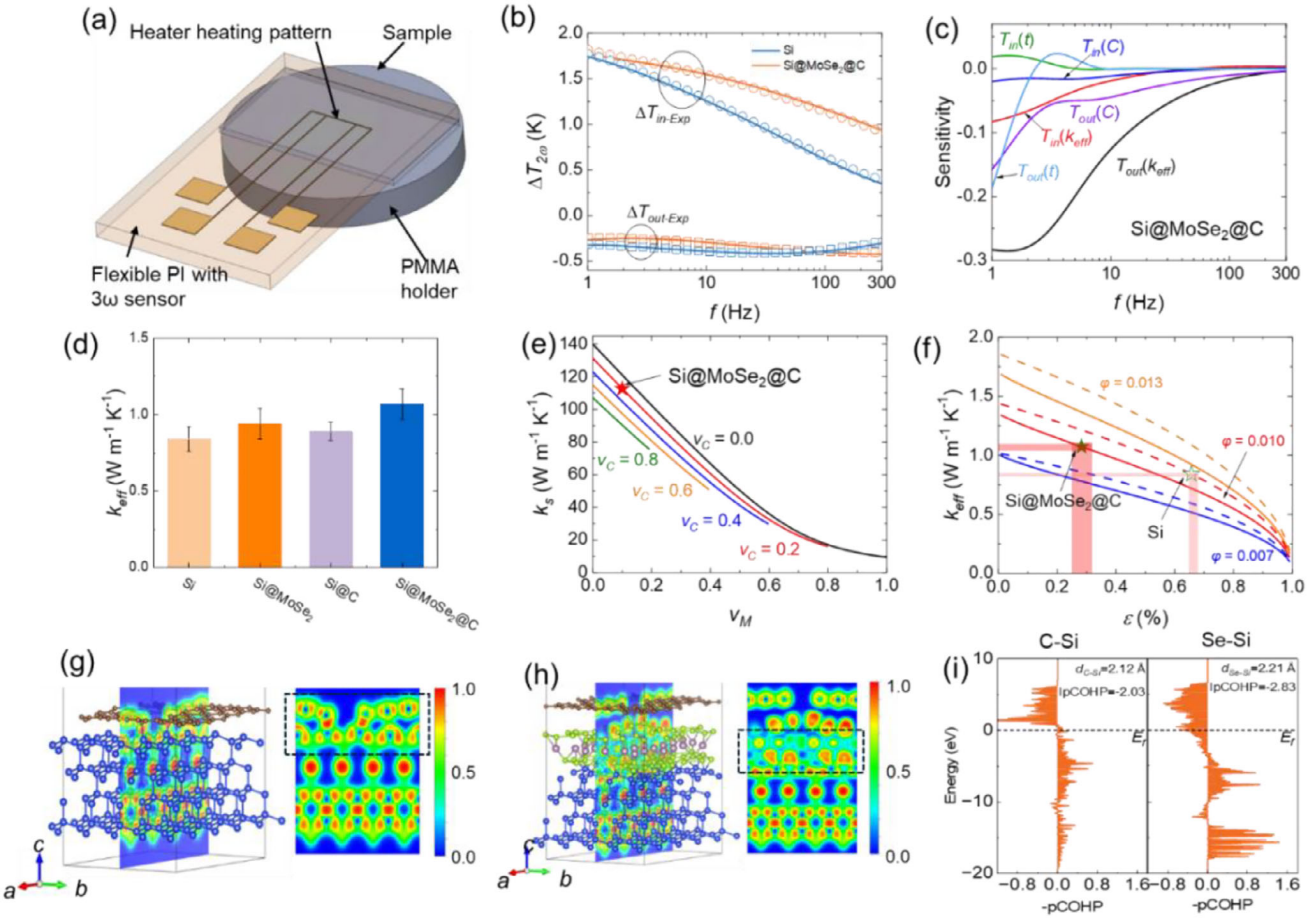
(a) Schematic of 3ω thermal measurement setup with Au heater/sensor on PI substrate. (b) Experimental data (symbols) with model fitting curves (solid lines) for Si and the Si@MoSe_2_@C─based matrix. (c) Sensitivity coefficients as a function of frequency for the Si@MoSe_2_@C─based matrix. (d) Comparison of *k* between the baseline Si, Si@MoSe_2_, Si@C, and the Si@MoSe_2_@C. Error bars reflect uncertainties obtained via standard error propagation that accounts for instrumental noise and parameter sensitivities [87]. (e) Thermal conductivity of the solid particle part (*k*_s_) in Si@MoSe_2_@C as a function of the volumetric fraction (*v*_M_) (see SI). (f) *k*_eff_ of Si (dashed lines) and Si@MoSe_2_@C─based matrix (solid lines) as a function of porosity (*ε*). (i) pCOHPs for the C─Si (left) and Se─Si (right) bonds.

Importantly, the higher‐potential features associated with the MoSe_2_ interlayer are beneficial in our LiPF_6_/EC–DEC electrolyte: by engaging the interface at ∼1.5–1.8 V, the thin, conductive MoSe_2_ layer homogenizes current distribution and templates an early, controlled interphase before deep Si lithiation (≲0.2 V), thereby lowering real‐cycle polarization and suppressing parasitic solvent reduction that otherwise onsets at lower potentials in additive‐free carbonates. This mechanistic role—MoSe_2_ as an electronically competent, ion‐permeable interlayer rather than a capacity contributor—is consistent with prior MoSe_2_‐on‐Si studies showing moderate‐potential interfacial engagement and stabilized cycling [20]. Such a mechanism is further supported by both DFT calculations and kinetic electrochemical measurements discussed in Sections 2.5 and 2.6. Specifically, DFT climbing‐image nudged elastic band (CI‐NEB) calculations (Figure 5i–l) show that the chemically bonded Si─Se─Mo heterointerface lowers the maximum Li^+^ migration barrier from ∼0.79–0.81 eV in Si@C to 0.61 eV in Si@MoSe_2_@C, Complementary DFT‐based pCOHP and ELF analyses (Figure 6g–i) further reveal stronger and more delocalized interfacial bonding in Si@MoSe_2_@C (Figure 6g–i). Experimentally, our galvanostatic intermittent titration technique (GITT, Figures S24g and S25) indicates an order‐of‐magnitude enhancement in the apparent Li‐ion diffusivity across much of the state‐of‐charge window, while electrochemical impedance spectroscopy (EIS, Figure S29) with distribution of relaxation times (DRT, Figure 5a–f) analysis quantitatively confirms that Si@MoSe_2_@C exhibits both the lowest charge‐transfer resistance and the most stable SEI resistance among all controls. These converging results explain the reduced polarization and improved reversibility observed in Figure 2a.

Galvanostatic charge–discharge profiles at 0.2 A g^−1^ in Figure 2b demonstrate the superior electrochemical behavior of Si@MoSe_2_@C compared to individual Si and MoSe_2_ anodes. To distinguish the contributions of Si and MoSe_2_, we provide a voltage‐segmented capacity analysis (Figure S10), where the low‐potential region (< 0.5 V vs. Li^+^/Li) corresponds to Si‐dominated alloying capacity and the higher‐potential features (>1.5 V) are associated with MoSe_2_ lithiation/conversion. Notably, the Si@MoSe_2_@C electrode exhibits both regions with markedly reduced polarization, consistent with improved interfacial kinetics. The relatively low reversible capacity of the porous‐Si control under our identical electrode formulation reflects the well‐known instability of high‐surface‐area nano/porous Si in carbonate electrolytes (rapid interphase growth and contact loss) [49], rather than a normalization artifact. As shown in Figure S10, potential‐resolved capacity analysis reveals that Si contributes approximately 47% of the total capacity at 0.2 A g^−1^, with the Si‐dominated region occurring below 0.5 V, indicating the MoSe_2_ layer primarily reduces kinetic losses rather than contributing high‐potential capacity. The composite electrode exhibits enhanced cycling stability, retaining a high specific capacity of 1054.6 mAh g^−1^ with 99.2% Coulombic efficiency (CE) after 100 cycles (Figure 2c). It is noted that the anode exhibits a high capacity exceeding the theoretical value for MoSe_2_, which is consistent with previous reports on nanostructured transition metal selenides and can be attributed to three established mechanisms: interfacial charge storage at metal/Li_2_Se interfaces formed during conversion, pseudocapacitive Li^+^ accommodation at edge/defect sites in MoSe_2_, and enhanced utilization of redox sites through improved electronic conductivity and stable heterointerfaces [50].

To elucidate component roles, we benchmarked pristine Si, MoSe_2_, Si@C, and Si@MoSe_2_ against Si@MoSe_2_@C (Figure S11). Pristine Si and MoSe_2_ show rapid capacity fading. Si@C enhances conductivity and buffers volume change yet still decays. The initial capacity of Si is not large, which is related to the particle aggregation due to the small size, which leads to poor utilization of active sites and accelerates capacity decay [51]. Si@MoSe_2_ benefits from chemically bonded interfaces that improve ion transport and stability, but both controls drop to ∼500 and ∼750 mAh g^−1^ by 40 cycles, respectively—well below Si@MoSe_2_@C. The Si@MoSe_2_@C architecture integrates a conductive, elastic carbon shell with a mechanically reinforcing, ionically favorable Si─MoSe_2_ interface, mitigating pulverization and contact loss and delivering superior capacity retention. These comparisons establish the hierarchy Si@MoSe_2_@C > Si@MoSe_2_> Si@C >individual components, demonstrating genuine synergy beyond additivity. SEM images (Figure 4) confirm that Si@MoSe_2_@C retains robust mechanical integrity after cycling, while Si@C shows partial structural collapse (Figure S12). This is further supported by TEM–EDS mapping (Figure S19) demonstrating intact Si/MoSe_2_/C architecture and by HRTEM/SAED (Figure S20) identifying preserved heterostructure with lattice fringes.

Rate capability testing further confirms the composite's robust performance, with capacity recovery to 1281.3 mAh g^−1^ upon returning from 1.0 to 0.2 A g^−1^, significantly outperforming both constituent materials (Figure 2d). Extended cycling evaluation demonstrates exceptional stability, with Si@MoSe_2_@C maintaining 782.7 mAh g^−1^ capacity and 99.5% CE after 400 cycles, corresponding to an ultralow capacity decay rate of only 0.03% per cycle, showing a performance far better than those of pure Si and MoSe_2_ (Figure 2e). The lower capacity observed at 1.0 A g^−1^ during rate‐performance testing compared to constant current cycling is attributed to insufficient equilibration time at high current densities, which limits ion diffusion and inadequate stabilization of the interface and SEI film, particularly given the volume changes in silicon particles that further restrict ion transport and reaction depth. In addition, the increase in constant current cycling capacity is also partly attributed to the continuous electrochemical activation process [52].

The radar chart in Figure 2f provides a holistic comparison of key performance metrics—including initial CE (ICE), rate capability, cycle number, and both initial and final capacity—between the Si@MoSe_2_@C anode and representative state‐of‐the‐art Si‐based anodes [53, 54, 55, 56, 57]. It highlights the outstanding and well‐balanced electrochemical performance of the Si@MoSe_2_@C composite across all evaluated parameters that outperforms various current Si anode technologies (see also Table S1), validating the synergistic benefits of the heterostructured composite design. Notably, our approach represents a fundamental departure from conventional strategies. While previous reports have focused on surface modification for SEI regulation or high‐temperature calcination for physical stability, our vacancy‐induced bonding mechanism creates chemically bonded heterointerfaces that simultaneously address multiple failure modes. The integration of surface modification strategies, high‐temperature stabilization techniques, and the vacancy‐induced bonding heterointerface presented here offers a rational pathway toward optimal Si‐based composite anodes that combine electrochemical excellence with enhanced thermal management—a critical requirement for next‐generation battery technologies [20, 33].

The Si@MoSe_2_@C electrode delivers an ICE of >80% (Figure 2c,f), which is competitive for porous Si‐based hybrid anodes tested in carbonate electrolytes, yet remains slightly below the near‐unity Coulombic efficiency obtained after the initial formation cycles. This first‐cycle inefficiency mainly originates from irreversible Li consumption associated with i) SEI formation on the highly accessible surface area of porous Si during the initial lithiation [58], and ii) the irreversible reduction of surface SiO_x_/Si–O species to electrochemically inactive Li_2_O and lithium‐silicate‐type phases that permanently trap Li inventory [59, 60]. Because the ICE penalty is fundamentally an electrode/cell‐level lithium‐inventory issue, it can be further mitigated by established practical strategies, such as replenishing lithium via prelithiation/sacrificial lithium sources and stabilizing the interphase through electrolyte optimization using functional additives [61, 62]. In this context, our vacancy‐induced chemically bonded heterointerfaces are primarily designed to suppress continuous interphase re‐growth and maintain high Coulombic efficiency during long‐term cycling, while the above lithium compensation strategies provide a complementary route to further raise the first‐cycle ICE for full‐cell implementation [62].

To assess practical applicability, the full‐cell performance of Si@MoSe_2_@C was evaluated using LiFePO_4_ as the cathode (Figure S13a). The Si@MoSe_2_@C anode was prelithiated to fully utilize the LiFePO_4_ capacity and to operationalize our evidence that a higher MoSe_2_ reaction potential is beneficial: by supplying Li in advance, the early interfacial engagement (including the ∼1.5 V feature) proceeds without borrowing Li from the cathode, suppressing any initial conversion‐related polarization of the MoSe_2_ interlayer, aligning the anode window with LiFePO_4_, and preserving practical voltage efficiency. The Si@MoSe_2_@C anode was pre‐lithiated to fully utilize the LiFePO_4_ cathode capacity. The LiFePO_4_||Si@MoSe_2_@C full‐cell displays a capacity of 125.2 mAh g^−1^ (on the basis of LiFePO_4_ cathode mass), which corresponds to an areal capacity of 1.1 mAh cm^−2^; while the ICE is as high as 82.7% (Figure S13b). In addition, the capacity remains 94.2 mAh g^−1^ after the current density increases from 0.1 to 0.5 A g^−1^ and finally returns to 0.1 A g^−1^. Notably, the full‐cell voltage profile in Figure S13a exhibits a broad plateau around ∼2.5–3.0 V, which is expected because the full‐cell voltage follows *V*_cell_ = *V*_cathode_  − *V*_anode_: although LiFePO_4_ shows a characteristic ∼3.4 V plateau vs Li/Li^+^ in half cells, the Si@MoSe_2_@C anode presents a sloping/hysteretic potential (with additional polarization under practical current), thereby shifting and smoothing the cathode plateau in the full‐cell curves rather than reproducing a sharp 3.3–3.4 V plateau. Rate capability testing reveals excellent capacity retention and reversibility at different current densities (Figure 2g). When the current density increases from 0.1 to 0.5 A g^−1^, the energy density decreases from 216.6 to 89.9 Wh kg^−1^ while the power density increases from 200.1 to 957.2 W kg^−1^ (Figure 2h). The full‐cell maintains 94.5 mAh g^−1^ after 100 cycles at 0.1 A g^−1^ with a CE exceeding 98% (Figure S13c). EIS reveals low charge transfer resistance (*R*_CT_), with impedance increase after cycling attributed to SEI formation (Figure S13d). The practical utility is demonstrated by powering an LED using the full‐cell (Figure S13e).

The versatility of Si@MoSe_2_@C was further demonstrated in all‐solid‐state lithium‐ion battery (ASSLIB) systems. The half‐cell ASSLIB, assembled with Si@MoSe_2_@C, the Li_6_PS_5_Cl (LPSCl) solid electrolyte, and Li–In alloy, achieves an initial areal capacity of 5.13 mAh cm^−2^ with 72.7% ICE (Figure S14a,b). Compared with liquid‐electrolyte LIBs, the Si@MoSe_2_@C anode exhibits distinct voltage profiles in ASSLIBs. We attribute this primarily to interfacial and compositional incompatibilities between MoSe_2_ and the sulfide electrolyte, which can alter reaction pathways, interphase formation, and overpotentials during charge/discharge. Despite these differences, the system exhibits high reversibility across different current densities and maintains > 98% CE after 50 cycles at 0.3 mA cm^−2^ with a capacity of 1.62 mAh cm^−2^ (Figure S14c,d). As shown in Figure S14e, the Nyquist plots deviate from the conventional semicircle and display a predominantly Warburg‐like, diffusion‐controlled line with a small high‐frequency knee. Such non–semicircle behavior is typical for sulfide‐based ASSLIBs where interfacial charge transfer is strongly coupled with solid‐state ion diffusion and distributed interfacial processes. The initial resistance (52.8 Ω) increases to 93.1 Ω after cycling, indicating the growth of an ionically conducting but resistive interphase and partial loss of intimate contact at the solid–solid interfaces [63].

Furthermore, a full cell ASSLIB was constructed using LiNi_0.8_Co_0.1_Mn_0.1_O_2_ (NCM811) as cathode material. The full cell configuration (Si@MoSe_2_@C||LPSCl||NCM811@LiNbO_3_) demonstrates a cycling stability with an initial areal capacity of 3.78 mAh cm^−2^ at 0.3 mA cm^−2^, while after 50 cycles the capacity remains 0.73 mAh cm^−2^ (Figures S14f,g, S15 and S16). The voltage plateaus of the ASSLIBs exhibit slight increases compared to liquid cells due to interfacial polarization that occurs at solid–solid interfaces, leading to voltage shifts [64]. These results confirm the application capability of Si@MoSe_2_@C across both conventional LIB and ASSLIB systems. The Si@MoSe_2_@C anode demonstrates superior performance in liquid electrolyte compared with all‐solid‐state configurations, which is ascribed to fundamental challenges in current solid‐state technology. The performance gap stems from lower intrinsic ionic conductivity of available solid electrolytes, formation of space charge layers at solid‐solid interfaces, potential interfacial reactions, and pressure‐dependent contact quality that impede ion transport kinetics relative to liquid systems [65, 66].

Notably, we present the LPSCl‐based all‐solid‐state cell as a proof‐of‐concept to evaluate the compatibility of the Si@MoSe_2_@C anode with sulfide solid electrolytes. Stable cycling with minimal impedance growth suggests that the chemically bonded heterointerface maintains ion transport and mitigates interfacial degradation (e.g., contact loss and parasitic interphase growth) commonly observed for Si in sulfide systems. However, the absolute capacity/rate performance remains constrained by solid‐state transport and contact limitations (such as the electrolyte‐network percolation, particle contacts, and pressure‐dependent interfacial resistance), reflecting the broader electrolyte/interface challenges for Si‐based ASSLIB [67, 68]. Crucially, our results establish Si@MoSe_2_@C as LPSCl‐compatible and positioned to benefit from advances in sulfide electrolytes and interfacial architectures (higher conductivity, optimized composite microstructures, engineered contact layers).

### 2.3 In Situ and Ex Situ Analysis of Reversible Phase and Bonding Evolution

To elucidate the reversible chemical and structural evolution of the Si@MoSe_2_@C anode during cycling, we combined in situ XRD and Raman with ex situ XPS/XRD to track phase evolution and interfacial stability. In the in situ XRD window (Figure 3a), the crystalline Si (311) reflection at 2*θ* ≈ 56.2° (Cu Kα) progressively weakens and disappears upon lithiation (discharge) [69], consistent with the well‐established amorphization of c─Si during initial lithiation, and reappears during delithiation. At deep lithiation (low‐voltage region approaching the cutoff), a weak diffraction feature appears at 2*θ* ≈ 39.4°. It disappears upon delithiation as the electrode reverts to an amorphous Li_x_Si/Si state at higher voltages, and is therefore reasonably assigned to Li‐rich crystalline Li─Si nanodomains (e.g., Li_15_Si_4_), which are known to form near full lithiation at low potentials (typically < 0.1 V vs. Li/Li^+^) [70].

The ex situ voltage‐dependent XRD patterns (Figure S17), collected over a wider 2*θ* range, capture the strong Si (111) reflection at 2*θ* ≈ 28.4° together with the MoSe_2_ (105) reflections; these peaks weaken during lithiation and recover to varying degrees after charging, supporting reversible Si dealloying and partial structural recovery of the composite [69]. This reversibility highlights the protective effect of the MoSe_2_ interlayer, which buffers stress through its layered structure and strong Si─Se─Mo covalent bonds, preventing irreversible pulverization. The overall lower diffraction intensity in Figure S17 (vs. the pristine powder patterns in Figure S3) is expected because the fabricated electrode contains amorphous carbon/binder that increases the background and dilutes the crystalline fraction. As deeply lithiated Li─Si phases are air/moisture sensitive [71], disassembly/transfer processing before ex situ scans can further suppress weak crystalline signals compared to the in situ results in Figure 3.

In situ Raman spectroscopy further captures the real‐time evolution of MoSe_2_ and carbon components in the electrode (Figure 3b,c). During discharge (lithiation), the out‐of‐plane *A*_1g_ mode of MoSe_2_ at 240 cm^−1^ weakens and redshifts, reflecting Li^+^ intercalation and associated changes in interlayer bonding strength. The intensities of the characteristic peak of Si at approximately 520 cm^−1^ gradually weaken, which is attributed to the formation of Li–Si alloy, while the intensities of the D and G bands of carbon (at 1337 and 1599 cm^−1^) decrease and shift to higher frequencies due to Li^+^ insertion into graphitic layers [72]. Upon charging, the peak intensities for both the MoSe_2_ *A*_1g_ mode and the carbon D and G bands recover, demonstrating excellent structural reversibility; the intensities of the Si peak also gradually increase with the progress of delithiation, indicating the recovery of Si in the electrode. This behavior is partly enabled by the heterostructure's stability, minimizing bond disruption [73].

Ex situ XPS provides atomic‐level insights into bonding evolution during the first cycle (Figure 3d–f). For Si 2p (Figure 3d), the fresh Si@MoSe_2_@C anode exhibits peaks at 99.1 eV (Si─Si) and 103.2 eV (Si─O). Upon full lithiation to 0.01 V (discharge), the Si peak shifts to 98.3 eV, reflecting alloying to form Li_x_Si. When the cell is subsequently delithiated (charged) to 0.32 V, the Li_x_Si peak intensity decreases with Si─Si peak reemerging as seen in the spectrum. The Si─O bond signal observed at full lithiation likely originates from a thin surface oxide on Si that persists and can partially reform due to slight exposure/transfer during measurements. For Se 3d (Figure 3f), characteristic peaks during lithiation at 1.74 V appear at 54.1 eV (Li_2_Se), 55.0 eV (Se 3d_5/2_), 55.4 eV (Se 3d_3/2_), and 56.3 eV (Se─O), indicating Li_2_Se formation from MoSe_2_ conversion. Upon delithiation to 2.2 V, the Li_2_Se peak weakens and shifts to lower binding energy, signifying reversible recovery of the original Se states [52]. Collectively, these analyses confirm that the heterostructure's covalent interfacial bonding promotes reversible reactions, laying the foundation for superior mechanical and electrochemical performance. In addition, the composite anode was analyzed by electron energy loss spectroscopy (EELS). The EELS spectrum of Si in the Si@MoSe_2_@C composite is shown in Figure S18. The Si L_2,3_ edge exhibits a characteristic feature near ∼106 eV, indicative of Si─O coordination [74]. These surface Si–O species promote oxygen‐vacancy formation and foster Si─Se─Mo bonding, enabling in situ construction of a tightly coupled Si@MoSe_2_ heterojunction. During the first lithiation, the partially oxidized layer can convert to Li_2_O, buffering volume change and stabilizing the interface, which benefits cycling stability [75].

### 2.4 Mechanical and Morphological Stability during Cycling

A critical advantage of the Si@MoSe_2_@C design is its enhanced mechanical stability, which effectively mitigates Si's extreme volume expansion (>300%) and minimizes pulverization, delamination, and interfacial failure, as substantiated by post‐cycling macro‐ and micro‐scale analyses. Optical microscopy and SEM highlight stark contrasts between pure Si and Si@MoSe_2_@C anodes (Figure 4). Before cycling, the pure Si anode exhibits a flat surface (Figure 4a_1_–a_3_), but after 100 cycles, it shows severe delamination from the current collector, large surface cracks, and thickness expansion from 9.8 to 29.7 µm (Figure 4b_1_–b_3_). In contrast, the Si@MoSe_2_@C anode remains robust, with no significant cracking, a smoother post‐cycling surface, and a moderate thickness increase from 10.1 to 17.2 µm (Figure 4c_1_–d_3_).

Three‐dimensional (3D) laser scanning optical microscopy further quantifies surface roughness changes (Figure 4e–h). Both anodes initially exhibit similar planar surfaces, with average roughness values of 32 nm for pure Si and 35 nm for Si@MoSe_2_@C. After 100 cycles, the pure Si anode's roughness surges dramatically to 139 nm (+107 nm) due to uncontrolled volume expansion (Figure 4h). In contrast, Si@MoSe_2_@C shows only a modest increase to 66 nm (+31 nm; Figure 4f), owing to the synergistic protection from the MoSe_2_ interlayer and carbon coating, combined with the porous Si core accommodating volume changes. These findings are supported by TEM after 100 cycles (Figures S19 and S20), which confirm the preserved core–shell architecture of Si@MoSe_2_@C. While post‐mortem TEM (Figure S15) shows some apparent particle agglomeration, part of this may arise from sample preparation artifacts (e.g., dehydration and beam effects); importantly, the hierarchical MoSe_2_@C shell provides physical spacing that suppresses direct Si─Si contact during cycling. Consistent with this interpretation, EDS elemental mappings (Figure S19b–f) show uniform distributions of Si, Mo, Se, C, and O, and an intact shell after 100 cycles, indicating robust interfacial integrity and effective mitigation of agglomeration‐induced performance losses. Overall, the MoSe_2_ and carbon layers provide effective physical spacing between adjacent silicon spheres, preserve buffer space for silicon's volume expansion, and maintain electrolyte percolation pathways, enabling stable cycling. SAED patterns and HRTEM images (Figure S20) further show intact lattice fringes with d‐spacings of 0.309 nm for Si (111) and 0.283 nm for MoSe_2_ (100), nearly identical to those of the as‐prepared sample (0.307 and 0.285 nm, respectively; Figure 1d,e), while the ∼7–10 nm carbon shell maintains its thickness and integrity, highlighting the heterostructure's resistance to mechanical degradation. Collectively, these results demonstrate that the Si@MoSe_2_@C design effectively prevents electrode collapse and maintains mechanical integrity, as schematically illustrated in Figure S21: while pure Si undergoes severe volume expansion leading to particle fracture and excessive SEI growth, the coated heterostructure buffers stress through its robust covalent Si─Se─Mo bonding, lattice matching, and encapsulating carbon layer, preserving particle morphology throughout cycling.

### 2.5 Kinetic Analysis, Impedance Spectroscopy, and DFT Insights Into Li^+^ Transport

Building on the structural and chemical insights from cycling analyses, we quantitatively evaluated the kinetic behavior and interfacial properties of the Si@MoSe_2_@C anode using CV, GITT, EIS, DRT analysis, and DFT calculations, revealing how the heterostructure enhances Li^+^ diffusion, lowers resistance, and suppresses polarization to bolster electrochemical stability. CV measurements at varying scan rates (0.1–0.5 mV s^−1^) quantify diffusion‐ and capacitive‐controlled contributions to kinetics, revealing the superiority of the heterostructure. For pure Si (Figure S22), capacitive fractions rise modestly from 26% to 43%, reflecting diffusion‐limited behavior due to volume expansion and low conductivity. Pure MoSe_2_ (Figure S23) shows similar limitations, with contributions increasing from 32% to 42% resulting from moderate ion accessibility. In stark contrast, the Si@MoSe_2_@C exhibits *b*‐values (power‐law exponents from the current–voltage relationship *i* = *av^b^*), ranging from 0.19 to 0.57 for oxidation peaks and 0.50 to 0.70 for reduction peaks, with capacitive contributions increasing from 32% to 63% (Figure S24a–f). The overlapping differential capacity curves over 30 cycles and robust current responses at lower scan rates confirm minimal polarization and rapid electron transfer kinetics. This capacitive dominance, moderated by the porous structure for balanced stability, stems from the heterostructure's optimized interfacial pathways, facilitating faster ion/electron transport and enhanced conductivity (see below).

To further quantify the diffusion kinetics, GITT measurements were performed for Si, MoSe_2_, and Si@MoSe_2_@C (Figures S24g and S25). The Li^+^ diffusion coefficient was calculated using Fick's second law: $D_{Li+}=\frac{4}{\tau \pi }\;{\left(\frac{n_{m}V_{m}}{s}\right)}^{2}{\left(\frac{\Delta E_{s}}{\Delta E_{\tau }}\right)}^{2}$, where *D*_Li+_ is the diffusion coefficient, *τ* is the relaxation time and Δ*E_s_* is the steady‐state voltage difference, and Δ*E_τ_* is the voltage change during the pulse time. As shown in Figure S24h,i, Si@MoSe_2_@C exhibits substantially higher *D*_Li+_ values compared to pure Si and MoSe_2_ anodes throughout both discharge and charge processes, demonstrating superior ion transport capabilities. This enhanced diffusion kinetics stems from optimized interfacial pathways that facilitate faster Li^+^ transport through engineered Si─Se─Mo bonding networks.

During discharge, the *D*_Li+_ behavior reveals complex transport mechanisms. As the potential decreases from 2.20 to 1.67 V, Si@MoSe_2_@C exhibits a distinctive non‐monotonic profile with an initial decrease followed by an increase in diffusivity, reaching 2.55 × 10^−9^ cm^2^ s^−1^. This behavior mirrors the trend observed in pure MoSe_2_ within the same voltage range, suggesting that the initial diffusion kinetics are dominated by Li^+^ intercalation into the MoSe_2_ layers with associated phase transitions. TheMoSe_2_@C coating suppresses volume expansion and produces higher stress states that may enhance Li^+^ transport behavior. As lithiation proceeds to lower potentials (< 1.67 V), *D*_Li+_ gradually decreases due to progressive Si–Li alloying reactions and the formation of more densely packed Li_x_Si phases.

During charge, all samples exhibit decreasing diffusion coefficients with increasing voltage, which may be attributed to stress‐diffusion coupling effects where volume contraction during Li^+^ extraction generates tensile stress that impedes ion transport [76]. The MoSe_2_@C coating provides enhanced mechanical constraint that facilitates structural stability throughout cycling. Importantly, Si@MoSe_2_@C maintains diffusion coefficients that are consistently one to two orders of magnitude higher than pure Si and MoSe_2_, demonstrating that the heterostructure's interfacial engineering provides superior transport pathways while ensuring mechanical integrity for good cycling stability.

Complementing the diffusion analysis, comprehensive resistance measurements reveal the exceptional electronic transport properties that distinguish Si@MoSe_2_@C from conventional architectures. In situ resistance measurements demonstrate that the heterostructure maintains remarkably low internal resistance throughout both lithiation and delithiation cycles (Figure S26). The internal resistance was quantified using Δ*R*_internal_ = Δ*U*_overpotential_/(*I*_current density_ × *M*_mass loading_), where the overpotential represents voltage deviation from equilibrium and mass loading normalizes resistance per unit active material. Single‐step GITT analysis reveals that Si@MoSe_2_@C exhibits significantly lower Δ*R*_internal_, Δ*E*_s_, and Δ*E*_τ_, values compared with the individual components during both charge and discharge processes (Figure S27), demonstrating the superior kinetic advantages of the heterostructure design.

In situ monitoring provides insights into the heterostructure's evolving transport properties during extended cycling. GITT measurements of Si@MoSe_2_@C across multiple cycles (20th, 50th, 150th, and 250th) show consistent voltage response profiles (Figure S28a). The reaction resistance evolution during discharge and charge processes across cycles (Figure S28b,c) remains within manageable ranges, suggesting that the Si@MoSe_2_ heterointerface maintains sufficient structural integrity to preserve reasonable transport properties over extended cycles.

EIS provides further quantitative evidence of these transport enhancements. Fresh cells exhibit charge transfer resistance (*R*_CT_) values of 819.5, 1180, and 2524 Ω for Si@MoSe_2_@C, Si, and MoSe_2_, respectively (Figure S29a). Remarkably, after 100 cycles, the *R*_CT_ of Si@MoSe_2_@C decreases to 358.6 Ω—the lowest among all samples—confirming enhanced interfacial conductivity [77] in our heterostructures after the stabilization of electrical transport channels (Figure S29b). In situ impedance monitoring during charge–discharge reveals that *R*_CT_ values increase slightly at 0.30–0.50 V during charging and become larger when reaching 2.70 V due to delithiation processes. During discharge, *R*_CT_ decreases significantly at approximately 1.50 V due to lithiation (Figure S30a,b). Critically, these impedance characteristics remain stable with minimal *R*_CT_ values even after 100 cycles (Figure S30c,d), which is attributed to the formation of a robust SEI film that ensures good cycling stability.

To deconvolute the individual resistance contributions and understand the fundamental kinetic advantages, DRT analysis [78] was employed to separate resistance components with distinct time constants: *R*_S_ (contact impedance), *R*_SEI_ (SEI transport impedance), and *R*_CT_ (Figure 5a,b). Each dynamic process is characterized by a specific time constant, enabling the precise identification of rate‐limiting steps. Before cycling, Si@MoSe_2_@C exhibits significantly lower impedances across all components compared to Si or MoSe_2_, directly attributable to enhanced Li^+^ diffusion facilitated by the heterostructure design (Figure 5a). After 100 cycles, Si@MoSe_2_@C maintains the lowest *R*_CT_ while simultaneously exhibiting the most stable *R*_SEI_, indicating formation of an optimal SEI layer that balances protection with transport efficiency (Figure 5b).

Dynamic DRT contour plots for Si@MoSe_2_@C during the first charge–discharge cycle in Figure 5c,d further reveal the cyclic evolution of each resistance component. The *R*_S_ peak (P1) remains stable throughout cycling, confirming excellent electrode‐collector contact integrity. The *R*_SEI_ peak (P2) exhibits systematic intensity changes that correlate with Li^+^ intercalation during charging and extraction during discharge. Most significantly, the *R*_CT_ peak (P3) demonstrates excellent reversibility with systematic increases during charging and decreases during discharge, indicating that Li^+^ transfer kinetics are intimately coupled to Li^+^ concentration gradients. After 100 cycles, these DRT signatures remain remarkably stable with only minor fluctuations, providing definitive evidence for the formation of a mechanically and electrochemically robust SEI film (Figure 5e,f).

To understand the superior transport properties of Si@MoSe_2_@C, DFT calculations were performed to map Li^+^ migration energy landscapes and reveal the atomic‐scale advantages of the heterostructure design. In our study, to build commensurate Si─Se─Mo interfaces, we matched the least‐common multiples of the in‐plane lattice vectors and performed supercell expansions with minimal in‐plane scaling, keeping the residual misfit within commonly accepted limits (approximately ≤ 5% for binary interfaces, with a slightly relaxed tolerance for the ternary heterostructure). Given the computational cost of CI‐NEB calculations, we selected the smallest commensurate supercells that achieved low misfit while retaining tractability. All models were fully relaxed for ionic positions (and along the out‐of‐plane lattice), with the in‐plane parameters constrained by the commensurate match. For conventional Si@C systems, Li^+^ diffusion from favorable surface sites toward inner layers induces Si atom displacements and local lattice distortion of the carbon (Figure 5g,h). While this deformation initially enhances Li^+^ surface adsorption, it creates prohibitively high diffusion energy barriers—reaching 0.79 eV at the surface and exceeding 0.81 eV for carbon skeleton penetration—due to strong Si─Li interfacial bonding (Figure 5i). These high barriers promote Li^+^ aggregation and structural instability, fundamentally limiting performance.

The critical advantage achieved by Si@MoSe_2_@C lies in the engineered heterointerface that creates optimized 3D ion transport channels. The heterostructure reduces the maximum Li^+^ diffusion energy barrier to 0.61 eV, while creating a gradual energy gradient that eliminates abrupt barrier peaks and transport bottlenecks (Figure 5j–l). This optimized energy profile stems from the formation of robust Si─Se─Mo bonding networks at the heterointerface, which fundamentally alter the local electronic environment and Li^+^ binding characteristics. The stress‐buffering effect of the heterostructure helps maintain these critical interfacial bonds during volumetric changes, preserving the favorable energy landscape throughout cycling. Quantitatively, this ∼ 24% reduction of the maximum Li^+^ migration barrier is directly reflected in the GITT‐derived diffusion coefficients (Figure S24h,i), where Si@MoSe_2_@C maintains *D*_Li+_ values one to two orders of magnitude higher than those of pure Si and MoSe_2_ over most of the state‐of‐charge window, and in the EIS/DRT results (Figure S29; Figure 5a,b), where Si@MoSe_2_@C consistently exhibits the lowest *R*_CT_ and the most stable SEI resistance. These kinetic advantages collectively account for the sharp low‐polarization redox peak observed in the CV curve of Figure 2a. This chemically bonded heterointerface design not only enhances structural integrity but fundamentally redefine Li^+^ transport pathways, representing a paradigm shift from conventional physical coating approaches to chemically bonded heterostructures that enable kinetic optimization.

### 2.6 Thermal Transport Enhancement Through Heterostructure Engineering and Electrochemical Performance at Elevated Temperature

We investigate how the Si@MoSe_2_@C heterostructure influences thermal transport properties, a largely unexplored factor critical for battery performance and safety that reflects interfacial contact quality, by employing the 3ω method [79, 80] to determine the effective thermal conductivity (*k*_eff_) of pellet matrices composed of Si@MoSe_2_@C, compared to pure Si particles prepared under identical conditions. This technique uses an Au strip (45 µm‐wide, 150 nm‐thick, 2 mm length) on a flexible PI substrate as both heater and sensor, with electrical insulation to prevent interference from conductive electrode materials while maintaining thermal contact with the sample pellet (Figure 6a). Temperature oscillations (Δ*T*) generated by controlled AC heating are analyzed across 1–300 Hz frequencies. The bi‐directional transfer matrix thermal model [81, 82, 83] (see Supporting Information) was employed to analyze the measured in‐phase and out‐of‐phase 3*ω* signals as a function of *f* with sample *k*_eff_ as the only fitting parameter, which provides excellent agreement with experimental data for both samples (Figure 6b). The measurement accuracy is validated through sensitivity analysis. For the Si@MoSe_2_@C‐based matrix (Figure 6c), the out‐of‐phase signal shows the highest sensitivity to the sample's *k*_eff_ among all parameters, where sensitivity $S_{\gamma }=\frac{\partial \ln\Delta T}{\partial \ln\gamma }\;$ and *γ* denote thermal properties or geometric parameters [84, 85], while maintaining minimal sensitivity to parasitic parameters such as sample thickness (*t*).

The thermal conductivity measurements reveal systematic improvements across the composite series (Figure 6d). The porous Si matrix exhibits a measured *k*_eff_ of approximately 0.84 W m^−1^ K^−1^, consistent with previously reported values for porous Si materials with comparable porosity levels [24, 86]. Both single‐component coatings improve thermal transport relative to bare Si (*k*_eff_ = 0.84 W m^−1^ K^−1^): Si@MoSe_2_ reaches 0.94 W m^−1^ K^−1^and Si@C reaches 0.89 W m^−1^ K^−1^. For Si@MoSe_2_, this improvement arises from the intrinsically favorable cross‐plane transport of MoSe_2_ and, critically, from chemically bonded Si─Se─Mo interfaces that reduce interfacial thermal resistance that is supported by our DFT analyses (Figure 6g–i with discussion below). The largest enhancement is observed for Si@MoSe_2_@C, where *k*_eff_ increases by ∼27% to ∼1.07 W m^−1^ K^−1^. All samples were prepared under identical conditions with the same material loading, coating content, and binder content to ensure a valid comparison.

To assess whether the improved heat‐transfer capability translates into enhanced electrochemical robustness under thermal stress, we compared the cycling behavior at 50°C of four electrodes (Si, MoSe_2_, Si@MoSe_2_, and Si@MoSe_2_@C). As shown in Figure S31, Si@MoSe_2_@C delivers the highest reversible capacity throughout 30 cycles and exhibits a slower fading trend: it retains 752 mAh g^−1^ at the 30th cycle, whereas Si, MoSe_2_, and Si@MoSe_2_ decrease to only 326–390 mAh g^−1^, respectively. Notably, from cycle 10 to 30, Si@MoSe_2_@C decreases from 1182 to 752 mAh g^−1^ (≈36% loss), while the control electrodes show a substantially larger relative loss over the same interval (48%–56%), evidencing a reduced decay rate at elevated temperature. This trend is consistent with the thermal‐conductivity results, supporting that the heterostructured composite—with strengthened interfacial coupling—helps mitigate temperature‐accelerated parasitic reactions and interphase growth.

To interpret the experimental observations, we employed a compact multiscale workflow. First, first‐principles phonon transport—combining DFT dispersions with an anharmonic Boltzmann transport equation (BTE) solver—provides the thickness‐dependent cross‐plane *k* of MoSe_2_. Next, the same phonon spectra feed a diffuse mismatch model (DMM) based on frequency‐resolved phonon transmission to obtain the interfacial thermal conductance (*G*) at Si/MoSe_2_ and MoSe_2_/C based on Equations S11 and S12 (see results below). Finally, these intrinsic *k* and interfacial *G* properties are propagated to the particle scale via a Bruggeman effective medium model and to the matrix scale via the Zehner–Bauer–Schlünder (ZBS) packed‐bed model, enabling a direct comparison with 3ω measurements.

To further understand the thermal performance differences between the two samples, we combined the Bruggeman model [88, 89] for the solid particle thermal conductivity (*k_s_*) of Si@MoSe_2_@C under effective medium approximation and a classical particle bed Zehner, Bauer, and Schlunder (ZBS) model [90, 91, 92] for the *k*_eff_ of the whole composite. *k*_s_ as a function of the MoSe_2_ volume fraction (*v*_M_) with different carbon volume fraction (*v_C_*), is shown in Figure 6e, while the model fitting of *k*_eff_ as a function of porosity *ε* and contact area ratio *φ* values for both samples is shown in Figure 6f. For both the Si and the Si@MoSe_2_@C‐based matrix, *φ* was found to be ≈0.01 despite the difference in porosity (*ε*). In the Bruggeman stage, the Si core conductivity is corrected to *k*_Si, real_ by series interfacial resistances using *G*_Si/MoSe2_ and *G*_MoSe2/C_ as shown in Equation S7b. The overall higher *k*_Si, real_ of Si@MoSe_2_@C compared to the baseline case of Si@C with much smaller *G*_Si/C_ enhance *k*_eff_, leading to the *k*_eff_ trend measured by 3ω.

The enhanced *k*_eff_ in Si@MoSe_2_@C compared to Si stems from multiple synergistic mechanisms. The primary enhancement originates from direct chemical bonding between hydrothermally grown MoSe_2_ and Si through Si─Se─Mo covalent bonds, followed by conformal carbon coating, improves interfacial thermal transport beyond what is achievable with purely physical (van der Waals) coatings. This chemically bonded heterostructure fundamentally improves interfacial thermal transport compared to conventional physical coatings limited by weak van der Waals interactions.

ZBS model analysis provides additional insights, showing that Si@MoSe_2_@C achieves lower porosity while maintaining similar contact area ratio compared to Si. Since the coating increases absolute particle size, maintaining a similar contact area ratio implies larger absolute contact areas and correspondingly reduced interfacial thermal resistance at each contact point. However, it is important to note that the ZBS model represents a highly idealized framework, and the non‐spherical particles cannot guarantee identical contact geometries despite similar contact area ratios. Nevertheless, the data support a combined mechanism of stronger chemical coupling and improved contact characteristics. Therefore, we attribute the enhanced *k*_eff_ in Si@MoSe_2_@C to the combined effects of improved interfacial bonding through vacancy‐assisted chemical coupling at the Si─Se─Mo interface and optimized contact characteristics that reduce the composite porosity. This enhanced thermal transport is critical for developing safe secondary batteries by addressing heat management challenges in high‐capacity Si anodes.

To investigate how Si─Se─Mo covalent bonding influences both thermal and electrical transport properties, we calculated the ELF [93] and pCOHP [94] (see details in Supporting Information). As shown in Figure 6g,h, the ELFs at the C─Si and Se─Si interfaces exhibit notable differences. The C─Si interface (Figure 6g) features highly localized regions with elevated ELF values, indicating a classic covalent bonding character between C and Si atoms. In contrast, the electron gas at the Se─Si interface (Figure 6h) is distributed over a wider area, reflecting a more “diffusive” electron gas. This behavior can be primarily attributed to the larger atomic radius and lower electronegativity of Se compared to C. Notably, such an extended electron gas distribution is expected to enhance phonon coupling across the interface, thereby effectively promoting interfacial thermal transport.

Figure 6i compares the −pCOHP profiles for the C─Si and Se─Si interfaces. In both cases, the predominantly negative −pCOHP indicates net bonding interactions. However, the Se─Si interface exhibits a notably larger negative area, consistent with stronger bonding. This is corroborated by the integrated pCOHP (IpCOHP): −2.21 eV for C─Si (bond length 2.12 Å) versus −2.83 eV for Se─Si (bond length 2.21 Å). The more negative IpCOHP for Se─Si signifies stronger covalent coupling at the Se─Si interface relative to C─Si, aligning with the improved interfacial phonon transmission observed experimentally. Such a strong bonding between MoSe_2_ and Si improves interface stability and promotes interfacial thermal transport. Our interface thermal conductance calculations in Figure S32, based on the DMM, reveal that *G*_Si/C_ = 52.75 MW m^−2^ K^−1^ for the baseline Si@C configuration commonly used in traditional Si anodes. This value is significantly lower than the interfacial conductances observed in the heterostructures, where $G_{\mathrm{MoS}e_{2}/C}$ = 256.79 MW m^−2^ K^−1^ and $G_{\mathrm{Si}/\mathrm{MoS}e_{2}}$ = 197.56 MW m^−2^ K^−1^. When accounting for the finite thickness of the MoSe_2_ interlayer by incorporating additional thermal resistance based on the cross‐plane thermal conductivity of MoSe_2_, the effective thermal conductance *G*_eff_ of the Si@MoSe_2_@C heterostructure remains superior to that of the baseline Si@C interface. Specifically, *G*_eff_ ranges from 85.11 to 72.54 MW m^−2^ K^−1^ as the MoSe_2_ thickness increases from 1 to 20 nm, demonstrating sustained thermal transport enhancement even with thicker interlayers. This combination of stronger and more delocalized Se─Si bonding (Figure 6i) and higher interfacial thermal conductance (Figure S32b) mitigates local overheating and stress accumulation during cycling, which in turn contributes to the stable and low‐polarization CV response and high CE of the Si@MoSe_2_@C anode.

## 3 Conclusions

In summary, we have developed a high‐performance Si@MoSe_2_@C anode through strategic heterointerface engineering to address the key challenges limiting silicon anode implementation. Oxygen vacancies generated via magnesiothermic reduction enable strong Si─Se─Mo covalent bonding between Si and MoSe_2_, forming chemically bonded heterostructures beyond conventional physical coatings that simultaneously improve mechanical stability, charge‐transport kinetics, and thermal management. The Si@MoSe_2_@C anode achieves a high specific capacity of 1054.6 mAh g^−1^ after 100 cycles, with excellent capacity retention over 400 cycles at 1.0 A g^−1^ with a low decay rate 0.03% per cycle, and CE exceeding 99.5%. Full‐cell demonstrations in both liquid electrolyte systems and ASSLIBs display high cycling efficiency, confirming wide applicability across next‐generation battery technologies. DFT‐based calculations reveal that the heterostructure reduces Li^+^ diffusion energy barriers by 24% through engineered 3D ion transport channels with quantitative pCOHP analysis confirming stronger covalent bonding at Se─Si interfaces. This work provides the first direct 3ω measurement of effective thermal conductivity in a siliconanode composite, increasing from porous Si (0.84 W m^−1^ K^−1^) to Si@MoSe_2_@C (∼1.07 W m^−1^ K^−1^) with a ∼27% enhancement, which we attribute to superior Si─Se─Mo interfacial thermal conductance. This work establishes chemically bonded heterointerface engineering as an effective route to high‐performance, mechanically robust, and thermally conductive rechargeable batteries, offering general design principles for next‐generation energy storage.

## 4 Experimental

### 4.1 Materials and Reagents

SiO_2_ nanospheres (Aladdin, 99.5%, diameter in 60–80 nm), magnesium powder (SCR, AR, 99.0%), hydrazine hydrate (SCR, AR, 50%), Na_2_MoO_4_·2H_2_O (Aladdin, AR, 99.0%), selenium powder (XING TA, AR, 99.95%), dopamine hydrochloride (Aladdin, AR, 98%), trihydroxymethyl aminomethane (Macklin, AR, 99%), and hydrochloric acid (SCR, AR, 36.0%–38.0%) were used directly without further purification.

### 4.2 Preparation of Porous Si Nanospheres

As‐received commercial SiO_2_ nanospheres were treated by using a magnesiothermic reduction method with a ratio of SiO_2_/Mg powders of 1:2. The mixture was heat‐treated at 650°C for 5 h in a hydrogen/argon (volume ratio = 5/95) mixed gas under a ramping rate of 5°C per min. After the reaction was completed, the samples were put in 1 m HCl solution for 3 h. Finally, the samples were washed with HCl solution and deionized water alternately for three times, and dried in an oven at 60°C for 24 h.

### 4.3 Preparation of Porous Si@MoSe_2_@C

Selenium powder (0.158 g) was dissolved in hydrazine hydrate (10 mL) under magnetic stirring to form solution A. Separately, Na_2_MoO_4_·2H_2_O (0.242 g) was dissolved in deionized water (20 mL) and sonicated for 15 min to form solution B. Solution A was slowly added dropwise to solution B in a 50 mL reaction vessel, followed by the addition of as‐prepared Si spheres (0.2 g) under continuous stirring for 30 min. The resulting mixture was transferred to a 50 mL autoclave and heated at 200°C for 24 h. The precipitate was collected, washed thoroughly with deionized water, and dried to obtain Si@MoSe_2_. For carbon coating, Si@MoSe_2_ (0.2 g) was dispersed in deionized water (100 mL) by sonication for 15 min. Tris(hydroxymethyl)aminomethane (2.42 g) was added to the dispersion, after stirring until completely dissolved, and the pH was adjusted to 8.0–8.5 using hydrochloric acid solution. Dopamine hydrochloride (120 mg) was then introduced, and the reaction proceeded for 24 h under continuous stirring. The resulting precipitate was collected, washed alternately with deionized water and ethanol, and dried in an oven at 60°C for 12 h. The samples used for thermal measurement were consistently mixed with hexagonal boron nitride powder (15 wt.%) as a pellet‐forming additive. Finally, the dried samples were subjected to pyrolysis at 550°C under nitrogen atmosphere for 3 h with a heating rate of 2°C min^−1^ to yield the Si@MoSe_2_@C composite.

### 4.4 Characterization

Morphology of the samples was analyzed on scanning electron microscopy (SEM, Hitachi S‐8100) and transmission electron microscopy (TEM, Hitachi HT‐7700). The phase and composition were characterized by X‐ray diffraction (XRD, Bruker D8 Advance), X‐ray photoelectron spectroscopy (XPS, ESCALAB 250), and Raman spectroscopy (Renishaw inVia confocal Raman microscope for both in situ and ex situ experiments). The elemental metal composition of the composite was quantified by inductively coupled plasma–optical emission spectroscopy (ICP–OES; Thermo Scientific iCAP PRO). The porosity of the samples was characterized by the BET analyzer (ASAP 2460). In‐situ XRD patterns were measured on a Rigaku SmartLab SE X‐ray diffractometer with in situ battery test accessories. The carbon content of the samples was analyzed using thermogravimetric analysis (Setaram Labsys Evo SDT Q600). The surface roughness of the electrode sheet was characterized by a 3D scan optical microscopy (ZEISS, SCAN TOP).

### 4.5 Electrochemical Tests

The active material, acetylene black, and polyvinylidene fluoride were mixed with a mass ratio of 7:2:1. The mixture was dispersed in N‐methylpyrrolidone. After magnetically stirring for 8 h, the slurry was coated onto the copper foil, and placed in a vacuum oven at 80°C. Coin cells were assembled in a glove box. The mass loading of each anode is Si@MoSe_2_@C (0.72 mg cm^−2^), Si (0.73 mg cm^−2^), Si@MoSe_2_ (0.71 mg cm^−2^), and MoSe_2_ (0.72 mg cm^−2^). The electrolyte contained LiPF_6_ with the volume ratio of ethylidene carbonate (EC) to diethyl carbonate (DEC) being 1:1. All comparative electrodes were fabricated with the same slurry composition and processing protocol. All specific capacities were calculated based on the mass of active material coated on the current collector, excluding the masses of the Cu foil, binder, and conductive additive. For the composite electrodes (Si@MoSe_2_@C, Si@MoSe_2_, and Si@C), the active‐material mass corresponds to the total mass of the composite powder in the electrode. For the single‐component control electrodes, the activematerial mass refers to the mass of Si or MoSe_2_, respectively. Electrochemical tests were conducted on the Neware battery system. For the charge–discharge measurements under high temperature, the cells were first kept at room temperature for 12 h, then transferred to a temperature‐controlled chamber (WBE‐SGD225L) connecting with Neware battery system by extended lines for another 12 h, before the tests were started. Cyclic voltammetry curves and electrochemical impedance spectroscopy spectra were measured using an electrochemical workstation (CHI 660E). In the full cell tests, the Si@MoSe_2_@C anode and LiFePO_4_ cathode were assembled using the same separator and electrolyte as those in the half cells. The cathode was prepared by mixing LiFePO_4_, acetylene black, and polyvinylidene fluoride in a weight ratio of 8:1:1 in N‐methyl‐2‐pyrrolidone. Before the full cell was assembled, the Si@MoSe_2_@C anode underwent 5‐cycles prelithiation treatment in a half‐cell, and then the full cell was assembled after disassembly. Approximately 120 µL of electrolyte was used for each cell, which was the same as that used in the half‐cells. The loading of LiFePO_4_ in the cathode is approximately 8.8 mg cm^−2^, with a thickness of 70 µm, while the mass loading of the anode is about 0.7 mg cm^−2^ with a thickness of 40 µm. The full cell was cycled at 0.1 A g^−1^ within a potential range of 1.5 to 3.8 V. The N/P ratio was 1.05. For all‐solid‐state battery tests, the anode consisted of Si@MoSe_2_@C and Li_6_PS_5_Cl mixed in a mass ratio of 1:1. A Li–In alloy counter electrode was prepared, including an In foil with a thickness of 100 µm and a Li foil (CANRD, AR, 99.95%). First, 80 mg of Li_6_PS_5_Cl was placed in a 10 mm diameter mold core and pressed at 300 MPa to form a solid‐state electrolyte. Then, 10 mg of the anode powders and Li–In alloy foil were respectively placed on both sides of the electrolyte and pressed at 70 MPa. All operations were performed in an Ar‐filled glovebox to ensure that the concentrations of H_2_O and O_2_ were below 0.1 ppm. The NCM811@LiNbO_3_ powder was mixed with Li_6_PS_5_Cl in a mass ratio of 7:3 in an agate mortar as the cathode for the Si@MoSe_2_@C||LPSCl||NCM811@LiNbO_3_ full cell. Charging and discharging at 0.2 A g^−1^ for 10 min followed by 2 h of rest was applied to collect the potential responses in different cycles during the galvanostatic intermittent titration technique measurements.

### 4.6 DFT Calculation

We employed Vienna ab initio simulation package (VASP) 6.1.0 [95, 96] to perform the spin‐polarized density functional theory (DFT) calculations within the generalized gradient approximation (GGA) using the Perdew–Burke–Ernzerhof (PBE) [97] formulation. We chose the projected augmented wave (PAW) potentials [98, 99] to describe the ionic cores and take valence electrons into account using a plane wave basis set with a kinetic energy cutoff of 400 eV. Partial occupancies of the Kohn−Sham orbitals were allowed using the Gaussian smearing method and a width of 0.05 eV. The electronic energy was considered self‐consistent when the energy change was smaller than 10^−6^ eV. A geometry optimization was considered convergent when the force change was smaller than −0.05 eV/Å^2^. Grimme's DFT‐D3 methodology [100] was used to describe the dispersion interactions among all the atoms. During structural optimization of the models, the 2 × 2 × 1 gamma‐point centered k‐point grid for Brillouin zone was used. Regarding the size of the DFT calculation model, the specific supercell parameters are: *a* = 15.0988 Å, *b* = 15.0988 Å, *c* = 35.0000 Å. For this large‐scale supercell system, a plane‐wave cutoff energy of 400 eV was employed, which is a well‐established value for first‐principles calculations of similar systems and ensures computational accuracy while maintaining efficiency [101, 102]. The choice of the K‐point grid was mainly determined by the size of the unit cell. Given that the dimensions of this system in the a and b directions were relatively large, a 2 × 2 × 1 Monkhorst–Pack grid was selected for the SCF self‐consistent calculation to provide adequate Brillouin zone sampling while avoiding unnecessary computational overhead from overly dense grids. Furthermore, the calculation of the diffusion barrier was carried out based on the most stable adsorption sites as determined through total energy comparison.

### 4.7 Thermal Conductivity Measurements

The 3ω thermal conductivity measurements were performed using a 45 µm‐wide, 150 nm‐thick Au strip (2 mm length) patterned on a 300 µm‐thick polyimide (PI) substrate. A 1.5 µm‐thick poly(methyl methacrylate) (PMMA) insulating layer was spin‐coated over the Au strip to electrically isolate the heater/sensor from the sample. Pellet samples (approximately 15 mm diameter, 300 µm thickness) were placed on the PMMA layer, and a constant pressure of 58 psi was applied using a weight to ensure good thermal contact between the sample and the heater/sensor. Sinusoidal AC current $I\left(t\right)=I_{0}\;\cos\left(\omega t\right)$ with *I*_0_ = 20 mA from a Keithley 6221 current source was applied to the heater/sensor to generate Joule heating and hence resistance oscillation at a frequency of 2*ω*, which generates sufficiently small temperature oscillations (< 2 K) in the sample across the 1–300 Hz frequency range for accurate thermal property determination. The corresponding electrothermal resistance oscillation, combined with the driving current at *ω*, resulted in a voltage oscillation at 3*ω* (*V*_3ω_), which was detected using a lock‐in amplifier (Stanford SR830). *V*_3ω_ was related to the temperature oscillation (*T*_2ω_) via the following expression: [79, 80]

(1) $$V_{3\omega }=\frac{1}{2}I_{0}R_{0}\beta T_{2\omega }$$

where, *β* was the temperature coefficient of resistance (TCR) of the heater/sensor at room temperature, which was experimentally calibrated after PMMA deposition onto the PI substrate, using a heating stage at a small current (0.5 mA). To extract *k_eff_* of the sample, we established the coordinate system: the *y*‐axis denoted the cross‐plane direction, normal to the sample/PMMA/PI interface, while *x*‐axis represented in‐plane direction. The experimentally measured in‐phase and out‐of‐phase component of *V*_3ω_ as a function of the current angular frequency ω  =  2π*f* were converted to complex amplitudes of *T*_2ω_, which were analyzed using a transfer matrix‐based bi‐directional multilayer heat conduction model: [81, 82, 83]

(2) $$T_{2\omega }=-\frac{p_{0}}{\pi }\int_{0}^{\infty }{\left(\frac{1}{G_{u}\left(\eta \right)}+\frac{1}{G_{d}\left(\eta \right)}\right)}^{-1}d\eta$$

(3) $$G_{u,d}=\frac{1}{k_{y,1}A_{1}\left(\eta \right)B_{1}\left(\eta \right)}\frac{\sin^{2}\left(b\eta \right)}{{\left(b\eta \right)}^{2}}$$

(4) $$A_{j-1}=\frac{A_{j}\frac{k_{y,j}B_{j}}{k_{y,j-1}B_{j-1}}-\tanh\left(B_{j-1}d_{j-1}\right)}{1-A_{j}\frac{k_{y,j}B_{j}}{k_{y,j-1}B_{j-1}}\tanh\left(B_{j-1}d_{j-1}\right)}$$

j=2,…,n,

(5) $$B_{j}=\sqrt{\varepsilon_{\mathrm{xy},j}\eta^{2}+\frac{i2\omega C_{j}}{k_{y,j}}}$$

(6) $$\varepsilon_{\mathrm{xy}}=\frac{k_{x}}{k_{y}}$$

(7) $$A_{n}=-\tanh{\left(B_{n}d_{n}\right)}^{s}$$

In these equations, *G*_u,d_(η) with subscript *u* and *d* denoting the thermal response functions for heat flow going upward and downward, respectively. The *p_0_* represented for the rms electrical heating power per unit length, *η* served as the integration variable, *i* represented the imaginary unit, and *n* denoted the total number of layers, including the sample, PMMA, and the PI substrate. The index *j* referred to the* j*‐th layer, counting from the topmost layer. Since the sample was isotropic, *k*_y,1_ was the desired *k*_eff_. *C* corresponding to the heat capacity of the sample obtained from the DSC characterization and density measurement (based on the mass and volume). The adiabatic boundary condition of the substrate was specified with *s* = 1 in our study.

### 4.8 Statistical Analysis

The images were processed on the Fireworks software (Adobe, San Jose, CA). The XRD, XPS, HRTEM, Raman, and electrochemical impedance spectroscopy (EIS) spectra were analyzed on Jade software (Materials Data, Livermore, CA), Peak Fit software (Systatsoftware, Inc., San Jose, CA), Digital Micrography software (Gatan Inc, Pleasanton, CA), Labspec software (Horiba, Kyoto, JP), OMNIC (Thermo Fisher Scientific Inc., Waltham, MA), and ZView (Scribner Associates, Inc., Charlottesville, VA), respectively. Experimental Results were analyzed on an OriginPro software (Origin Lab, Northampton, MA). DFT calculations were performed on Vienna ab initio simulation package (VASP) 6.1.0.

## Conflicts of Interest

The authors declare no conflicts of interest.

## Supporting information




**Supporting File**: advs74165‐sup‐0001‐SuppMat.pdf.

## Data Availability

The data that support the findings of this study are available from the corresponding author upon reasonable request.
