# Supplementary material for: A Si‐MoSe2 Heterostructured Anode with Enhanced Thermal Transport and Electrochemical Performance for Liquid and All‐Solid‐State Lithium‐Ion Batteries
Source: Adv Sci (Weinh). 2026 Jan 31;13(20):e23320. doi: 10.1002/advs.202523320 (PMC13067772; doi:10.1002/advs.202523320)
Supplement: Supplementary file 1 — Supporting File: advs74165‐sup‐0001‐SuppMat.pdf. [file ADVS-13-e23320-s001.pdf]

Supplementary information

A Si-$MoSe_2$ Heterostructured Anode with Enhanced Thermal Transport and Electrochemical Performance for Liquid and All-Solid-State Lithium-Ion Batteries

Yajun Zhu[1,2,3], Jiaqi Gu[4], Guangwu Zhang[4], Tianli Han[1], Yang Lu[4], Zhongbing Li[3], Hang Su[3], Fei Wang[3], Haojun Xu[1], Wentuan Bi[2,*], Qiye Zheng[4,*], Jinyun Liu[1,*]

1. Key Laboratory of Functional Molecular Solids, Ministry of Education, College of Chemistry and Materials Science, Anhui Normal University, Wuhu, Anhui 241002, PR China

2. Institute of Energy, Hefei Comprehensive National Science Center, Hefei, Anhui 230031, PR China

3. Anhui Deeiot Energy Technology Co., Ltd, Wuhu, Anhui 241002, PR China

4. Department of Mechanical and Aerospace Engineering, The Hong Kong University of Science and Technology, Hong Kong SAR, PR China

*Correspondence and requests for materials should be addressed to bwin@ustc.edu.cn (Wentuan Bi); qiyezheng@ust.hk (Qiye Zheng) and jyliu@iim.ac.cn (Jinyun Liu).

Effective medium and ZBS model: The k of the crystalline Si particles in both samples is assumed to be the 140 W m^{-1} K^{-1}.[1] The anisotropic k of $MoSe_2$ respectively and those for BN are also taken from the literature.[2,3] The in-plane and out-of-plane *ks* of graphitic C are taken as 2054 and 5.5 W m^{-1} K^{-1}, respectively.[4,5] The average k (k_a) for these anisotropic materials is calculated as $k_a = \sqrt{k_{in}k_{out}}$,[6] where k_{in} and k_{out} represented the in-plane and out-of-plane thermal conductivity, respectively. The mean k of the Si@$MoSe_2$@C solid particle (k_s) could be estimated using the Bruggeman model considering the volume fraction of added $MoSe_2$ (v_M), C (v_C) and BN (v_{BN}):[7]

$$\sum_{i-1}^{n} v_i \frac{k_i - k_s}{k_i + 2k_s} = 0, \qquad (S1a)$$

where v_i is the volume fraction of component i, and k_i is the thermal conductivity of component i (i = Si, $MoSe_2$, C or BN). k_s is the *effective* conductivity of the composite particles of Si@$MoSe_2$@C or other baseline cases (e.g., Si@$MoSe_2$ and Si@C) with the fixed fraction of the BN binders included.

Interfacial resistances at Si/$MoSe_2$ and $MoSe_2$/C reduce the Si core conduction via a series correction for a particle of diameter d_{Si}:

$$k_{Si,real} = d_{Si}\left(\frac{d_{Si}}{k_{Si,real}} + \frac{2}{G_{MoSe_2@C}} + \frac{2}{G_{Si@MoSe_2}}\right)^{-1} \qquad (S1b)$$

This equation estimates the conductivity of the Si particles in the real composite. $k_{Si,real}$ then replaces k_{si} in the effective medium model of Eq. (7a). The values of the $G_{MoSe_2@C}$ and $G_{MoSe_2@C}$ are calculated using the first-principles based diffuse mismatch model (DMM) given in Eq. 18 below. This contact resistance correction is incorporated in the calculation for Fig. 6 in the main text.

For the matrix compressed particles in our study, the classical particle bed heat transfer model based on the Zehner, Bauer, and Schlünder (ZBS) model[8-10] is used to analyze the thermal transport and estimate the contact area ratio using the porosity and measured k_{eff} of the Si@$MoSe_2$@C-based matrix. The porosity (ε) of Si and Si@$MoSe_2$@C-based matrixes is determined by combining Brunauer–Emmett–Teller (BET) measurements and the measured sample density, yielding values in the range of 64.9%–67.8% and 25.1%–32.1%, respectively.

The ZBS model then relates the 3ω measured k_{eff} to the

$$\frac{k_{eff}}{k_g} = (1-\sqrt{1-\varepsilon})\varepsilon[(\varepsilon - 1 + k_G^{-1})^{-1}] + \sqrt{1-\varepsilon}[\varphi k_p + (1-\varphi)k_c], \tag{S2}$$

where k_g is the thermal conductivity of the air, taken as 0.026 W m^{-1} K^{-1},[11] φ is the contact area ratio, k_p is the thermal conductivity ratio between Si@$MoSe_2$@C and air (k_s/k_g), and k_c is the reduced core thermal conductivity in ZBS model, as defined by the following equation:

$$k_c = \frac{2}{N}\left(\frac{B}{N^2}\frac{k_p - 1}{k_p k_G}\ln\frac{k_p}{B[k_G + (1-k_G)k_p]} - \frac{B+1}{2} - \frac{B-1}{N k_G}\right) \tag{S3}$$

The factor N and deformation factor (B) are given by

$$N = \frac{1}{k_G}\left(1 - \frac{B k_G}{k_p}\right) - B\left(\frac{1}{k_G} - 1\right), \tag{S4}$$

$$B = C_f\left(\frac{1-\varepsilon}{\varepsilon}\right)^{\frac{10}{9}}, \tag{S5}$$

where C_f is the shape factor with a value of 1.25 for spherical particles. The ZBS model accounted for the Knudsen effect in gas-filled voids by means of the parameter:

$$k_G = \left(1 + \frac{l}{d}\right)^{-1}, \tag{S6}$$

where d is the average equivalent sphere diameter of the Si@$MoSe_2$@C particles with a value of 300 nm measured by scanning electron microscopy (SEM) and l is the modified mean free path of the air molecules with a value of 38.5 nm at 300 K and 1 atm.[12]

Calculation of the interface thermal conductance in composites materials: We employ the diffuse mismatch model[13,14] (DMM) to quantify the interfacial thermal conductance (G) and systematically investigate the effect of different interfaces on thermal transport (see below). The $G_{Si/C}$ is evaluated as 52.75 MW/m^2/K, which is much lower than the $G_{MoSe_2/C}$ (256.79 MW/m^2/K) and $G_{Si/MoSe_2}$ (197.56 MW/m^2/K). To quantify the effective G (G_{eff}) between C and Si after $MoSe_2$ insertion, we used the following formula to calculate the G_{eff} variation to quantify the role of the contact resistance:

$$\frac{1}{G_{eff}} = \frac{1}{G_{MoSe_2@C}} + \frac{l}{k_{MoSe_2}} + \frac{1}{G_{Si@MoSe_2}}, \tag{S7}$$

where $\frac{1}{G_{MoSe_2@C}}$ and $\frac{1}{G_{Si@MoSe_2}}$ are the resistance of the two additional interfaces. As $MoSe_2$ has different thicknesses (l) in the sample as shown in Fig. 1(d), which will impact the thermal conductivity (k_{MoSe_2}) significantly. Due to the lack of experimental studies on the cross plane k as a function of the thickness in $MoSe_2$, we calculate the thickness-dependent k_{MoSe_2} based on first-principles Boltzmann transport equation method using the Fourphonon[15] package, in which the k-mesh was set to 15 × 15 × 9. We obtained the harmonic, third-order, and fourth-order anharmonic force constants of $MoSe_2$ through the finite difference method[15,29] based on the 3 × 3 × 1 supercell[19]. The phonon dispersions and group velocities in are calculated using Phonopy[29] package. The displacement amplitude employed in the finite difference method is set to 0.09 Å, which can reduce the numerical noise in the computation of soft interlayer force components.[19] The third-order and forth-order force constants are obtained by taking into account the fifth and third nearest neighboring atoms, respectively. For the thickness (d) dependent k_{MoSe_2}, the boundary scattering rates (τ_b) were evaluated by assuming that the phonon mean free path is capped by a structural length scale l, yielding $\tau_b = v/l$,[30] which represents fully diffuse scattering.[31,32]

As shown in Fig. S31a, the k_{MoSe_2} increases with increasing thickness, and our bulk k_{MoSe_2} agrees well with experimental values[16] and other theoretical studies[17–19]. It can be seen from Fig. S31b that the G_{eff} changes from 85.11 MW/m^2/K to 72.54 MW/m^2/K with l rising from 1 nm to 20 nm, which is much higher than $G_{Si/C}$ (52.75 MW/m^2/K). Therefore, inserting $MoSe_2$ between C and Si forms Si-Se-Mo bonds, which helps improve thermal transport performance.

Electron localization function (ELF) calculation: The electronic local density[20] (ELF) is a quantitative measure of the probability of finding an electron in the vicinity of a reference electron with the same spin, and it ranges from 0 to 1. An ELF value close to 1 indicates strong electron localization (such as in covalent bonds or lone pairs), a value around 0.5 corresponds to electron-gas-like behavior, and values near 0 indicate delocalization. The ELF is defined as

$$ELF = \left[1 + \left(\frac{D}{D_h}\right)^2\right]^{-1}, \tag{S8}$$

where D is the curvature of the conditional pair probability density of the actual system, and

D_h is that of a homogeneous electron gas with the same density. This function effectively distinguishes between different types of chemical bonding and electron pair distributions. We performed ELF calculations using the electronic density and wave functions obtained by density functional theory (DFT) calculations in VASP[21,22]. The VESTA[23] package was used for post-processing and visualization of ELF results.

Projected Crystal Orbital Hamilton Population (pCOHP) calculation: The pCOHP[24] provides an energy-resolved description of the bonding and antibonding contributions between the selected atomic pairs, which is particularly useful for identification and clarification of nature and strength of chemical bonds within the material. The pCOHP is defined as

$$\mathrm{pCOHP}_{ij}(E) = H_{ij}(E) \cdot D_{ij}(E), \tag{S9}$$

where $H_{ij}(E)$ is the Hamiltonian matrix element between atomic orbitals i and j, and $D_{ij}(E)$ is the corresponding projected density of states at energy E. $D_{ij}(E)$ is expressed as

$$D_{ij}(E) = \sum_{n,\mathbf{k}} c_{i,n\mathbf{k}}^{*} c_{j,n\mathbf{k}} \delta(E - \epsilon_{n\mathbf{k}}), \tag{S10}$$

where coefficients $c_{i,n\mathbf{k}}$ and $c_{j,n\mathbf{k}}$ are the projections of the Bloch wave function for band n at k-point $\mathbf{k}$ onto atomic orbitals i and j, respectively, with $c_{i,n\mathbf{k}}^{*}$ being the complex conjugate of $c_{i,n\mathbf{k}}$ The term $\epsilon_{n\mathbf{k}}$ denotes the energy eigenvalue of band n at $\mathbf{k}$, and $\delta(E - \epsilon_{n\mathbf{k}})$ is the Dirac delta function, which ensures that only the electronic states with energy equal to E contribute to the sum. The summation runs over all bands n and all k-points in the Brillouin zone. To make the graphical analysis and comparison of bond strengths more straightforward, we use the negative pCOHP (-pCOHP) in our analysis. The positive -pCOHP indicates bonding interactions, while negative -pCOHP corresponds to antibonding interactions. This makes the graphical analysis and comparison of bond strengths more straightforward. By integrating the pCOHP up to the Fermi level, we obtain the IpCOHP, which serves as a quantitative measure of bond strength. The pCOHP calculations were performed using the LOBSTER[25] based on the wavefunction files generated by VASP[21,22].

Diffuse mismatch model (DMM)

For a given interface between two materials 1 and 2, the diffuse mismatch model (DMM) assumes that all phonons undergo elastic scattering[14,26–28]. The vibration transmission

coefficients at the interface are given by

$$\zeta^{1\to2}(\omega) = \frac{\sum_\lambda [\boldsymbol{q}_{\lambda,2}(\omega)]^2}{\sum_\lambda [\boldsymbol{q}_{\lambda,2}(\omega)]^2 + \sum_\lambda [\boldsymbol{q}_{\lambda,1}(\omega)]^2}, \tag{S11}$$

where $\zeta^{1\to2}$ denotes the vibration transmission from side 1 to side 2, $\boldsymbol{q}$ is the wave vector, ω is the frequency of the mode λ. The vibration transmission can now be used to calculate the interfacial thermal conductance, which is given by

$$G = \frac{1}{8\pi^2} \sum_\lambda \int_{\boldsymbol{q}_{\lambda,1}>0} \hbar\omega_{\lambda,1}(\boldsymbol{q}_{\lambda,1}) \boldsymbol{q}_{\lambda,1}^2 \, \zeta^{1\to2} \times \left| v_{\lambda,1}^{\perp}(\boldsymbol{q}_{\lambda,1}) \right| \frac{\partial f}{\partial T} d\boldsymbol{q}_{\lambda,1}, \tag{S12}$$

where $v_{\lambda,1}^{\perp}(\boldsymbol{q}_{\lambda,1})$ is the group velocity normal to the interface, f is the equilibrium vibration distribution at the temperature T, i.e., the Bose-Einstein distribution.

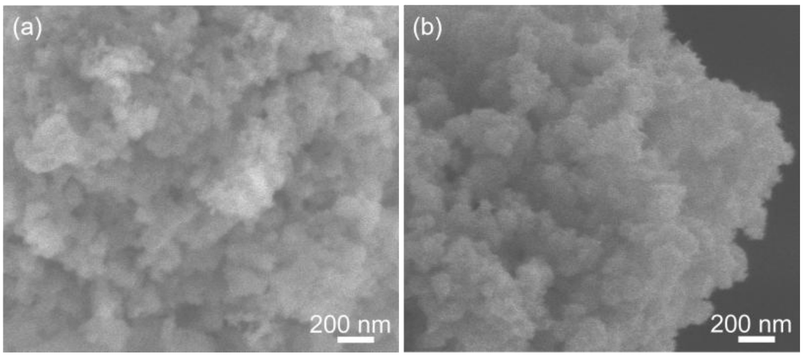

Fig. S1 SEM images of pure (a) Si and (b) $MoSe_2$.

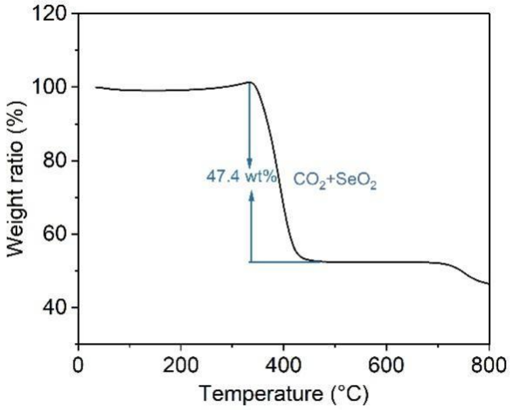

Fig. S2 TGA curve of Si@$MoSe_2$@C.

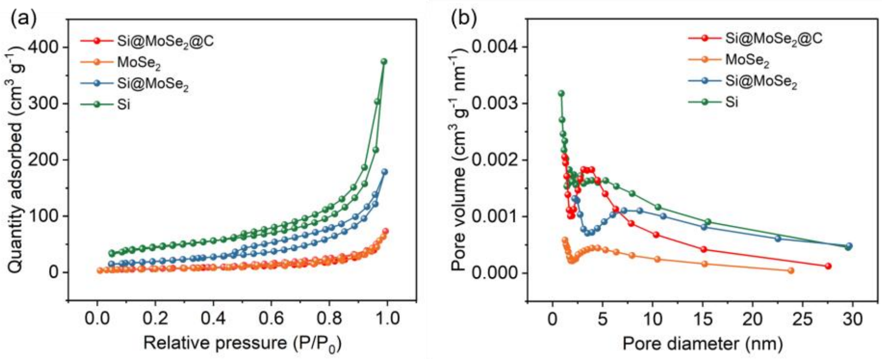

Fig. S3 (a) The N_2 adsorption-desorption isotherms and (b) pore-size distribution of Si@$MoSe_2$@C, $MoSe_2$, Si@$MoSe_2$, and Si.

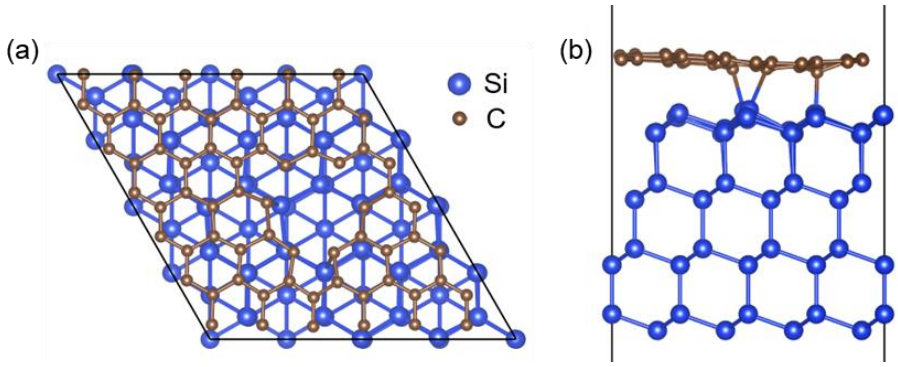

Fig. S4 (a) Top- and (b) side-view images of the Si@C.

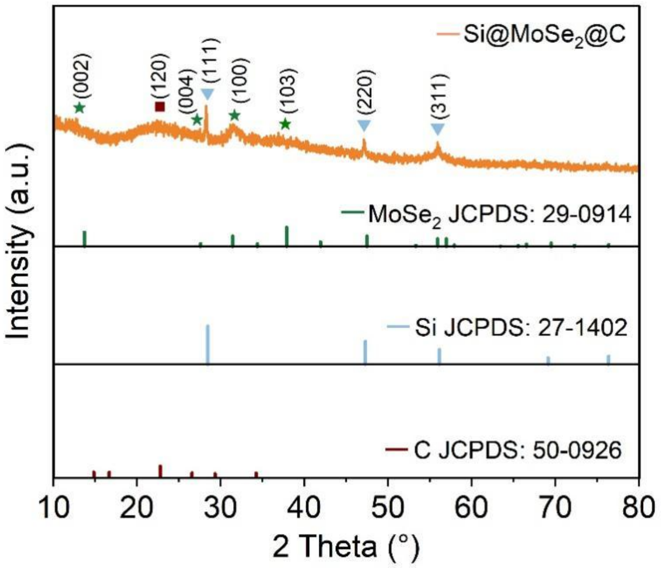

Fig. S5 XRD pattern of Si@$MoSe_2$@C.

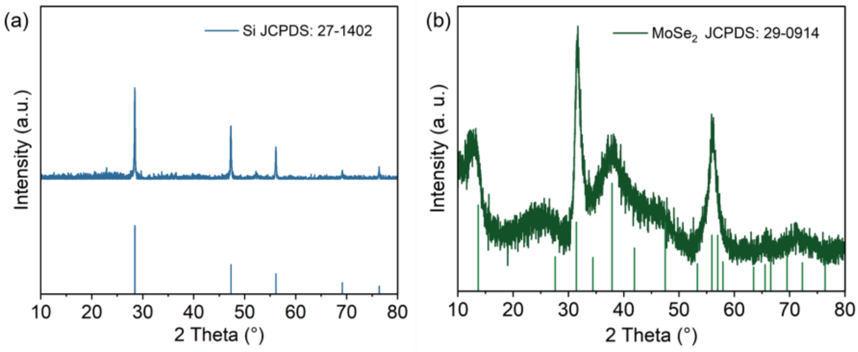

Fig. S6 XRD patterns of pure (a) Si and (b) $MoSe_2$.

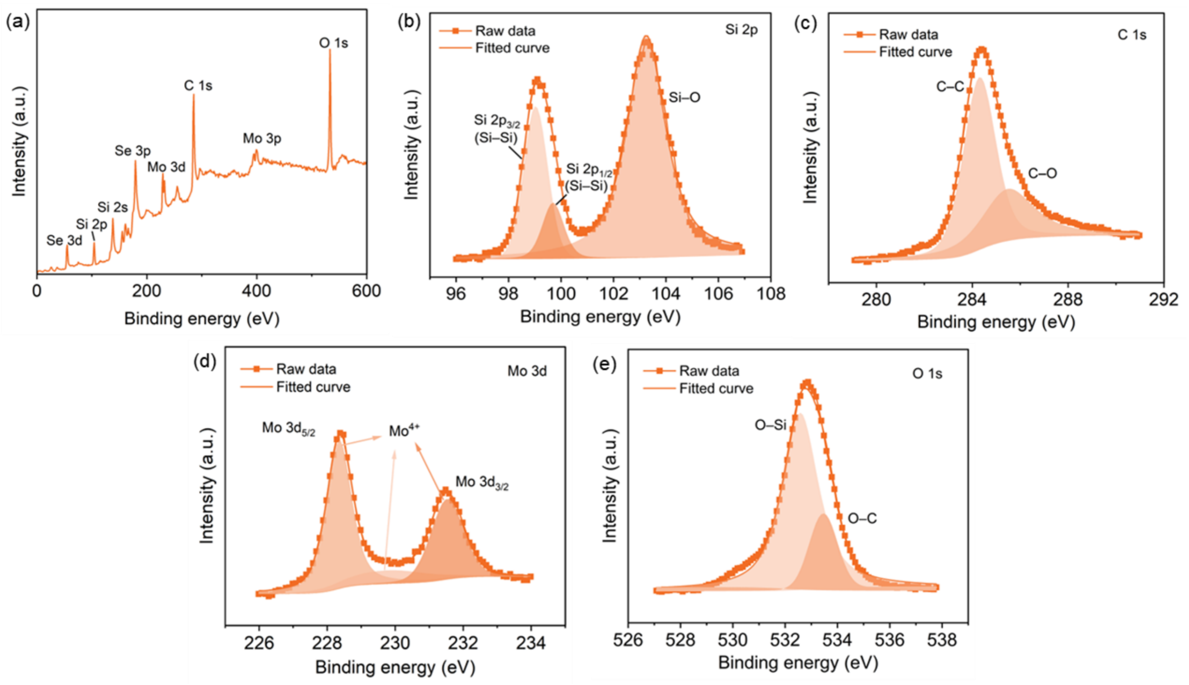

Fig. S7 XPS spectra of Si@$MoSe_2$@C: (a) survey spectrum, (b) Si 2p, (c) C 1s, (d) Mo 3d, and (e) O 1s.

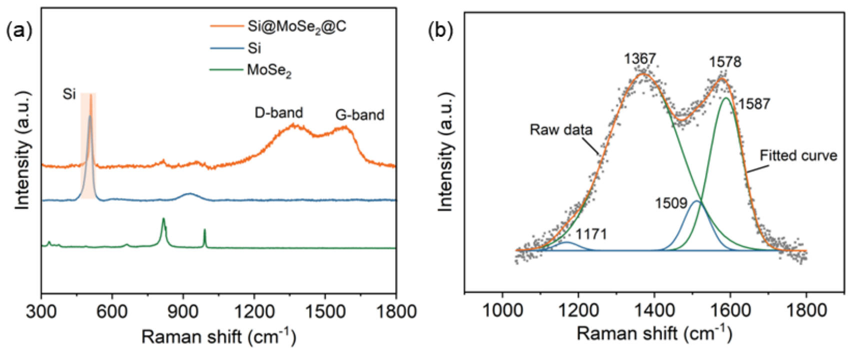

Fig. S8 (a) Raman spectrum and (b) the deconvoluted spectrum of Si@$MoSe_2$@C at the D and G bands.

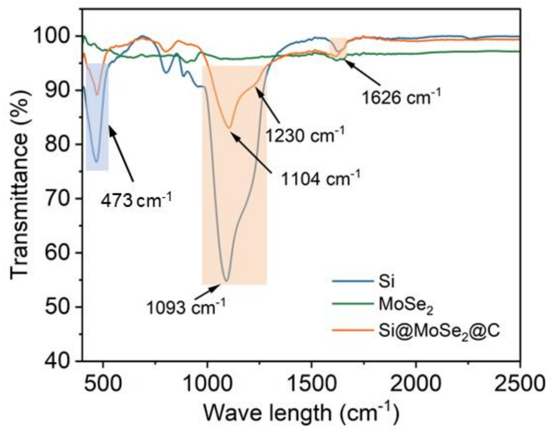

Fig. S9 Fourier transform infrared (FTIR) spectroscopy spectra of Si@$MoSe_2$@C, $MoSe_2$, and Si.

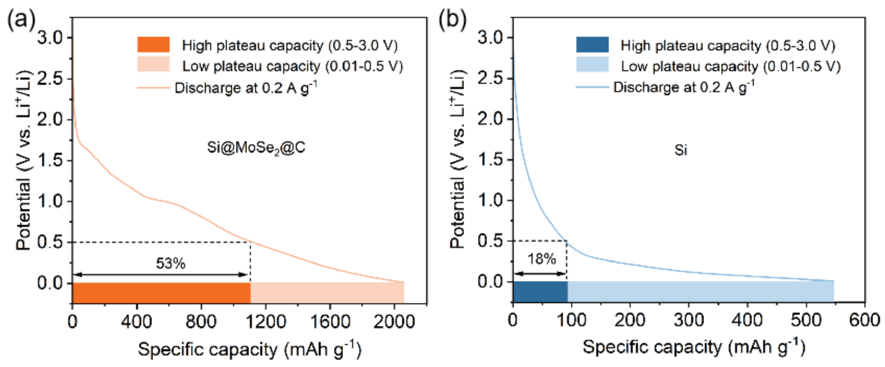

Fig. S10 Low- and high-potential capacities of the (a) Si@$MoSe_2$@C and (b) Si.

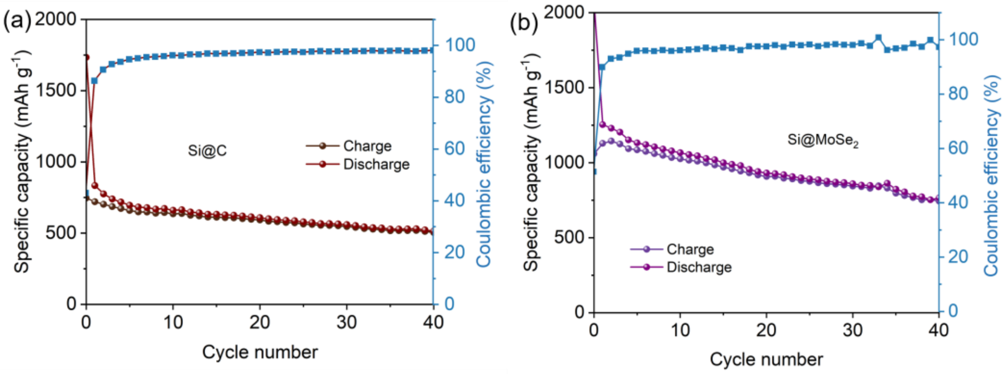

Fig. S11 Cycling performance of the (a) Si@C and (b) Si@$MoSe_2$ at 0.2 A g^{-1}.

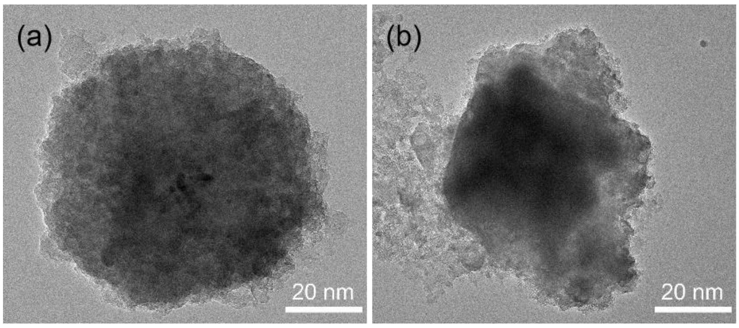

Fig. S12 TEM images of Si@C (a) before and (b) after 100 cycles at 0.2 A g^{-1}.

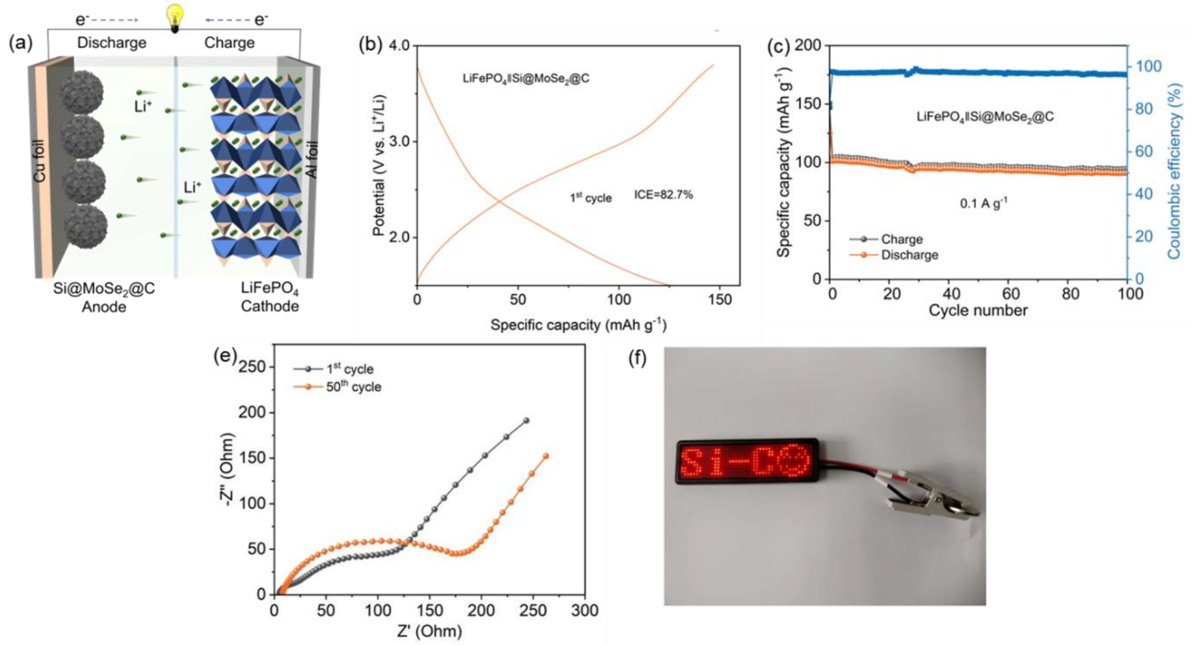

Fig. S13 (a) Schematic illustration of the LFP||Si@$MoSe_2$@C full cell. (b) Typical charge/discharge curve of the full cell at 0.1 A g^{-1}. (c) Cycling performance of the full cell at 0.1 A g^{-1}. The full cell capacities were calculated on the basis of weight of $LiFePO_4$. (d) EIS of the full cell at the 1st cycle and 50th cycle. (e) Digital image of LFP||Si@$MoSe_2$@C full cell lighting an LED circuit board.

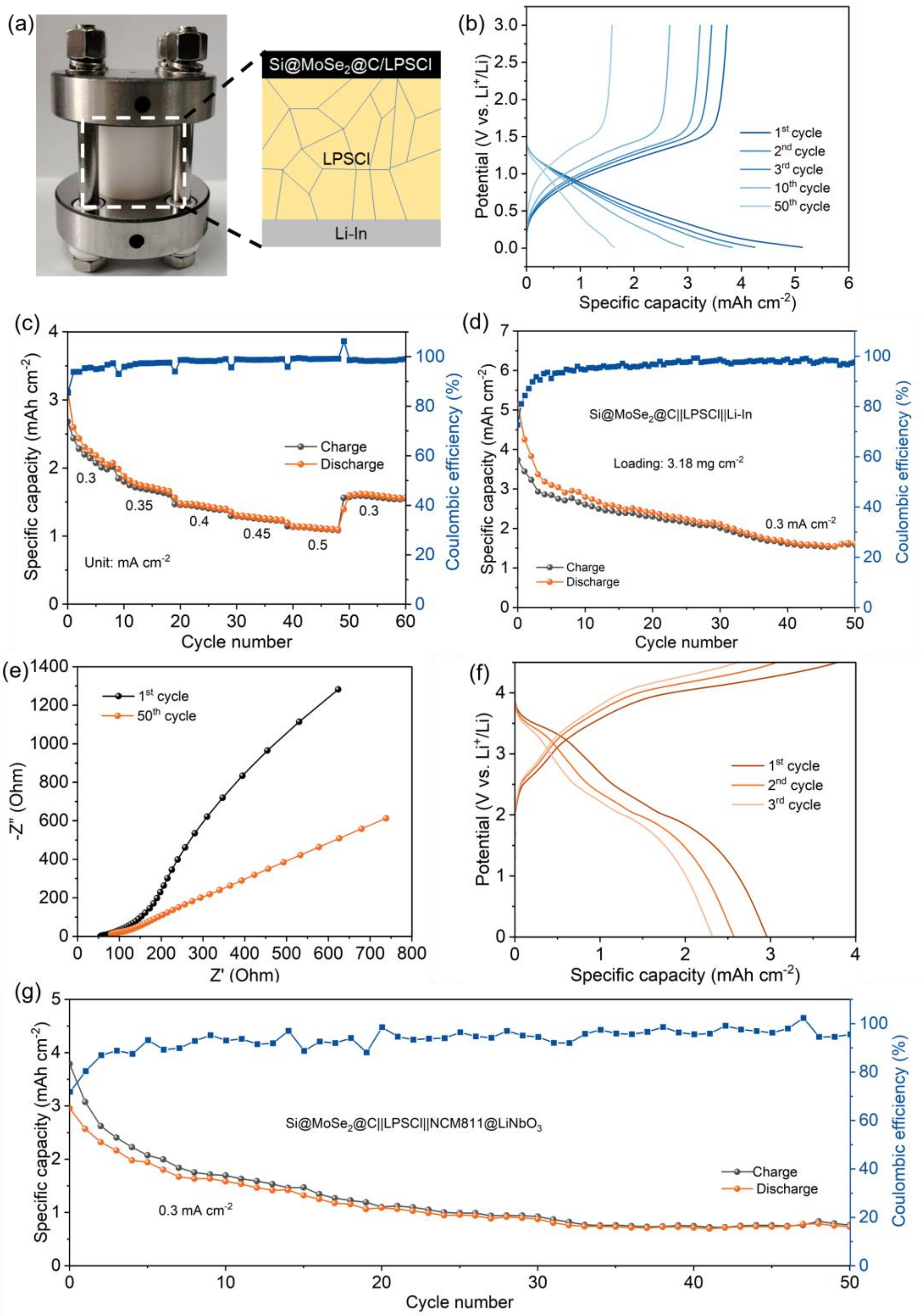

Fig. S14 Electrochemical performance of ASSLIBs with a Si@MoSe$_2$@C anode. (a–e) Half-cell: schematic, galvanostatic profiles at 0.3 mA cm^{-2}, rate performance, cycling stability, and EIS at the 1st and 50th cycles. (f–g) Full-cell (Si@MoSe$_2$@C||LPSCl||NCM811@LiNbO$_3$): galvanostatic profiles and cycling.

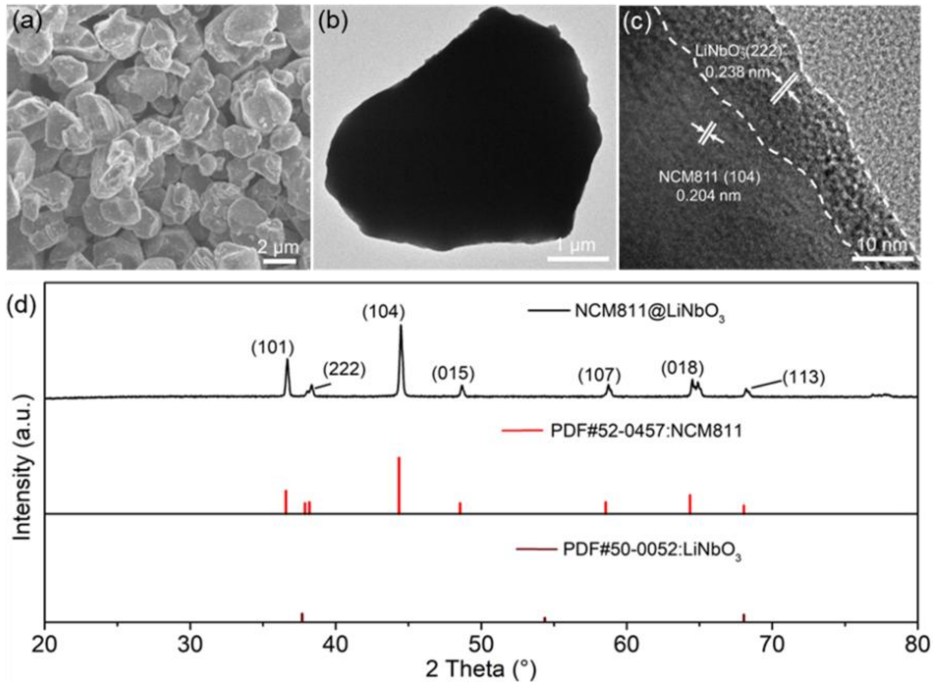

Fig. S15 (a) SEM, (b) TEM and (c) HRTEM images, and (d) XRD pattern of NCM811@$LiNbO_3$ as cathode material.

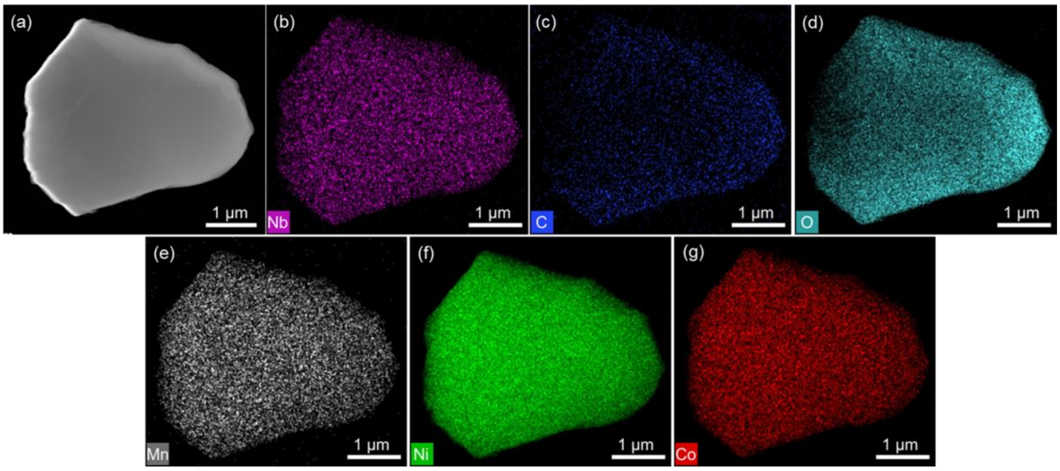

Fig. S16 (a) TEM and (b-g) mapping images of the NCM811@$LiNbO_3$.

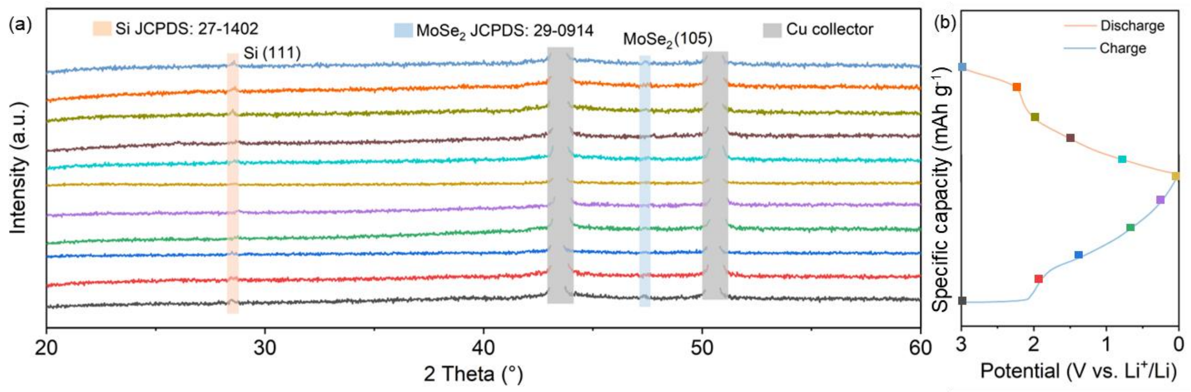

Fig. S17 (a) The *ex-situ* XRD patterns of Si@MoSe$_2$@C during cycling. (b) Corresponding cycling curves illustrating each potential. The grey regions are signals from Cu current collector.

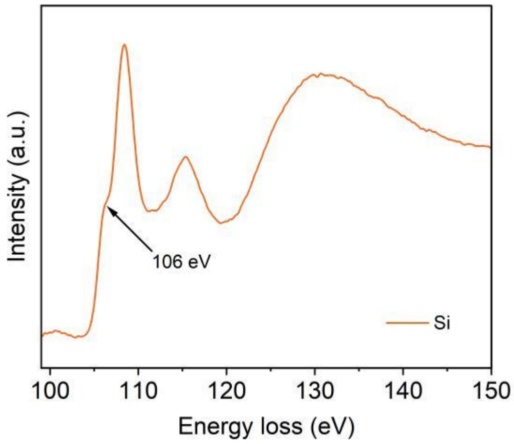

Fig. S18 EELS spectrum of the Si@MoSe$_2$@C for Si.

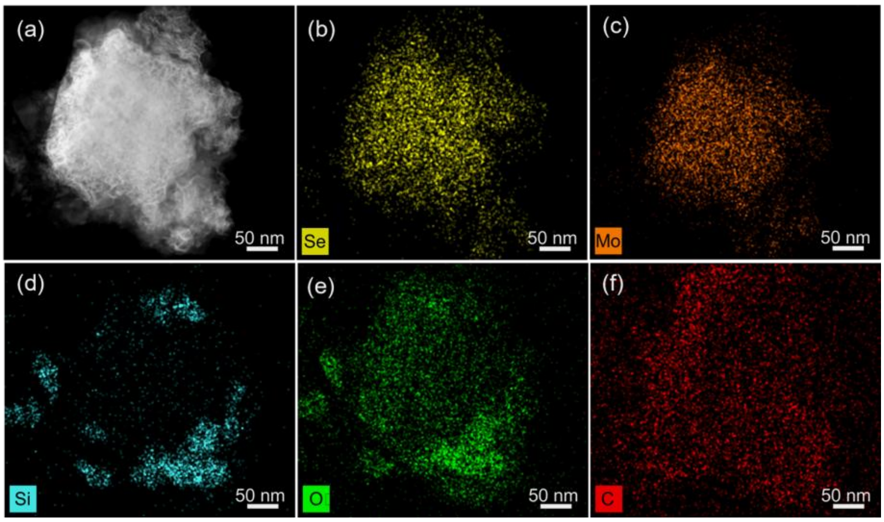

Fig. S19 (a) TEM and (b-f) elemental mapping images of Si@MoSe$_2$@C after 100 cycles at 0.2 A g^{-1}.

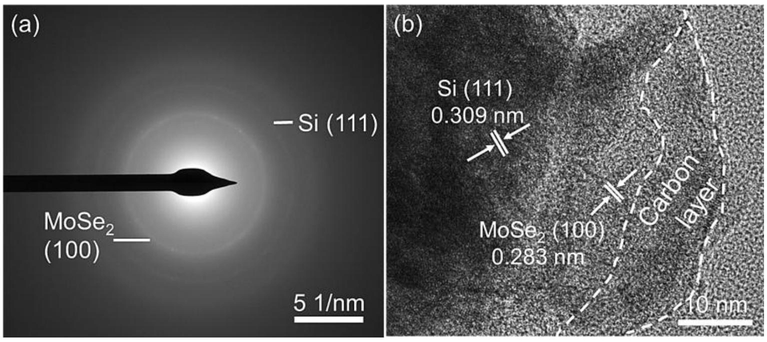

Fig. S20 (a) SAED pattern and (b) HRTEM image of Si@MoSe$_2$@C after cycling 100 times at 0.2 A g^{-1}.

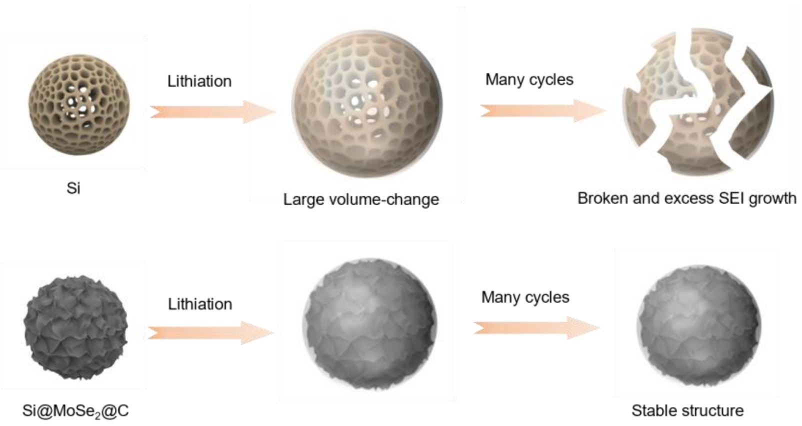

Fig. S21 Schematic diagram of the volume-change of Si and Si@MoSe$_2$@C during the long-term cycling process.

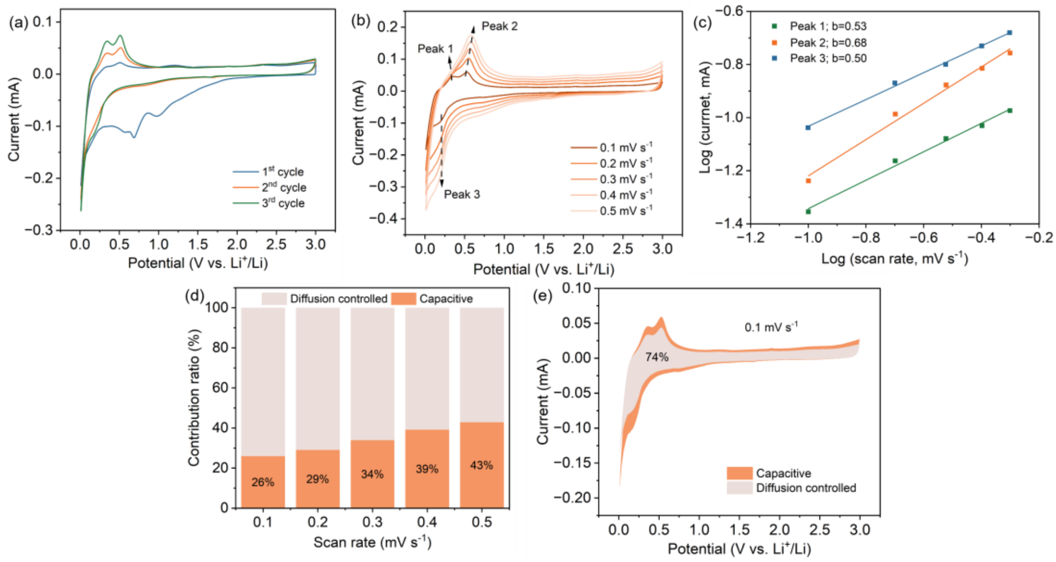

Fig. S22 CV curves of Si at (a) 0.1 mV s^{-1} and (b) different rates of 0.1-0.5 mV s^{-1}. (c) Fitted curves of log(i) vs. log(v). (d) Ratios of capacitive and diffusion-controlled contributions. (e) Pseudo-capacitive response of Si at 0.5 mV s^{-1}.

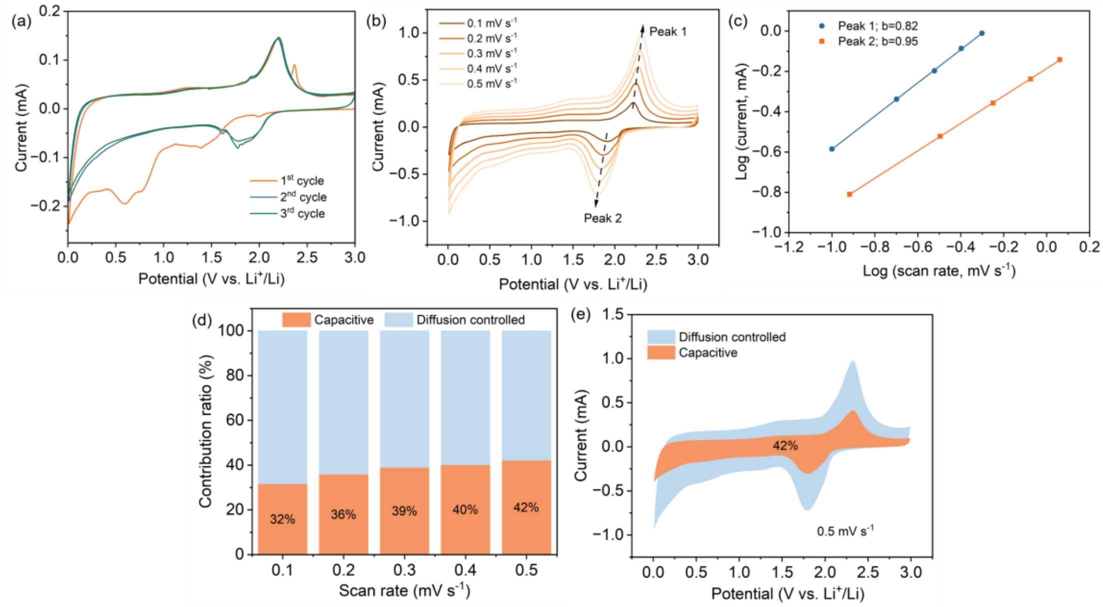

Fig. S23 CV curves of $MoSe_2$ at (a) 0.1 mV s^{-1} and (b) different rates of 0.1-0.5 mV s^{-1}. (c) Fitted curves of log(i) vs. log(v). (d) Ratios of capacitive and diffusion-controlled contributions. (e) Pseudo-capacitive response of $MoSe_2$ at 0.5 mV s^{-1}.

CV Curves of Si@MoSe$_2$@C, Si and MoSe$_2$ at Varied Scan Rate

The CV curves of Si@MoSe$_2$@C were investigated from 0.1 to 0.5 mV s^{-1} (Fig. S24a). Fig. S24b presents the contour plot in the CV mode. As the scanning rate decreases, the anode exhibits an obvious current response, which is related to the rapid electron transfer kinetics. As shown in Fig. S24c, the differential capacity curves for the first 30 cycles overlap well, demonstrating a tiny polarization. According to the formulas for peak current (i) and scan rate (v): $i = av^b$ and $\log(i) = b\log(v) + \log(a)$, the b values of the oxidation peaks are 0.19, 0.29, and 0.57, respectively, while the b values of the reduction peaks are 0.50 and 0.70. The contributions of corresponding capacitive control (k_1v) and diffusion control ($k_2v^{0.5}$) are determined by the formula $i = k_1v + k_2v^{0.5}$. The results present when the scan rate increases, the capacitive contribution gradually increases (Fig. S24d and e). When the scan rate is 0.5 mV s^{-1}, the capacitive contribution ratio is approximately 63% (Fig. S24f). This indicates that the electrochemical behavior is predominantly capacitive-controlled, with the porous structure moderating the rapid capacitive response for enhanced stability[33]—significantly outperforming pure MoSe$_2$ and Si (Fig. S22 and S23).

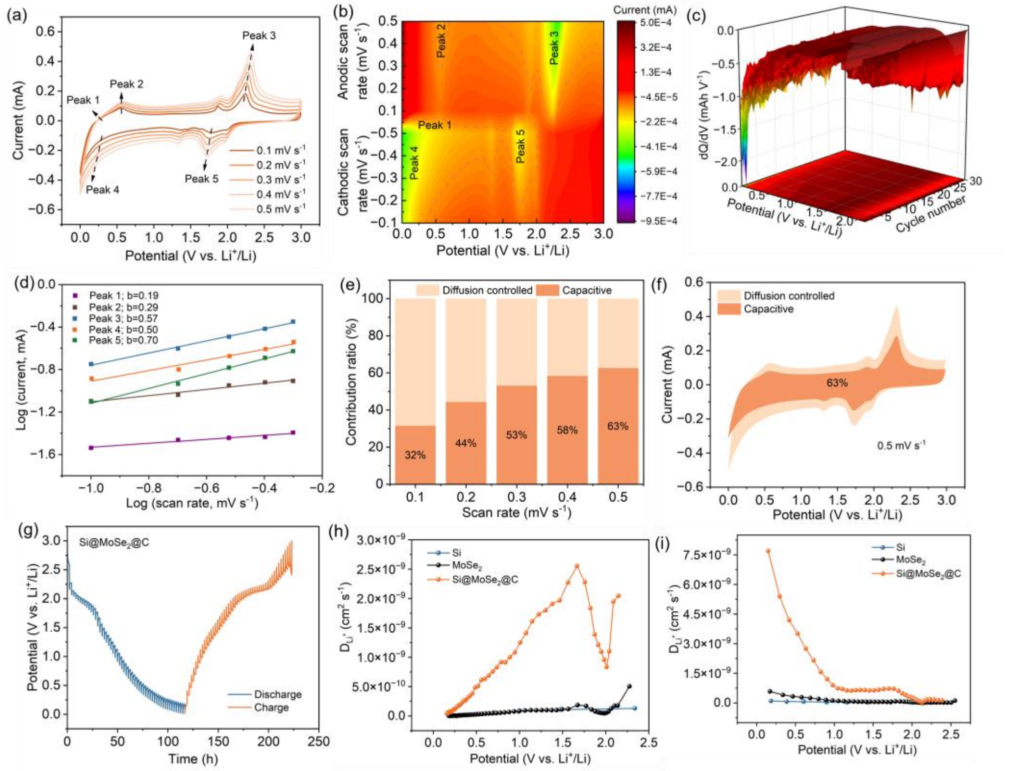

Fig. S24 (a) CV curves of Si@MoSe$_2$@C cycling at 0.1-0.5 mV s^{-1}. (b) Contour plot of CV curves at different scan rates. (c) 3D dQ/dV plot of differential capacity for the first 30

cycles. (d) Fitted curves of log(i) vs. log(v). (e) Capacitance and diffusion control contributions. (f) Pseudocapacitive response of Si@$MoSe_2$@C at 0.5 mV s^{-1}. (g) Voltage distribution under GITT. Ion diffusion coefficients of Si, $MoSe_2$, and Si@$MoSe_2$@C during (h) discharge and (i) charge.

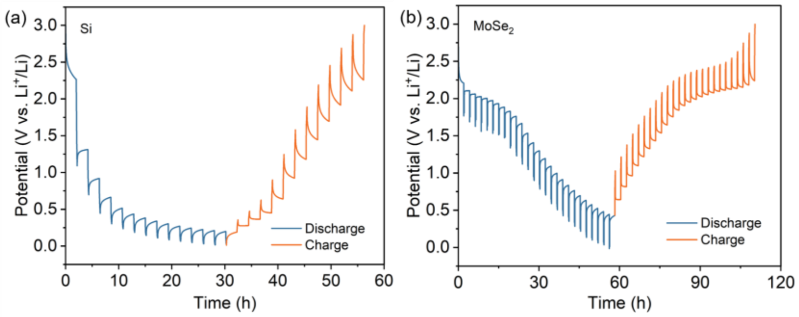

Fig. S25 GITT time-potential distributions of (a) Si and (b) $MoSe_2$.

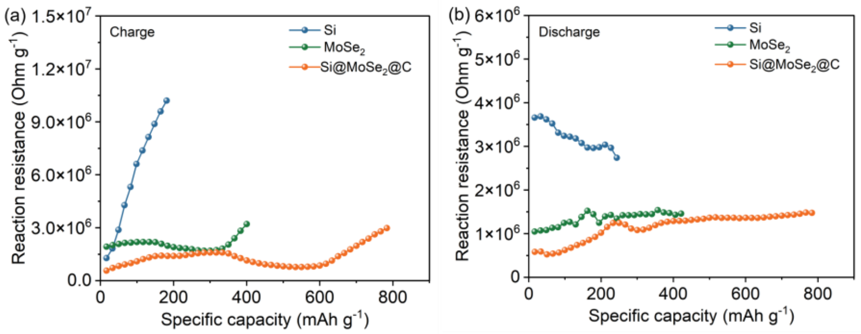

Fig. S26 *In-situ* reaction resistances during (a) charge and (b) discharge of Si, $MoSe_2$, and Si@$MoSe_2$@C.

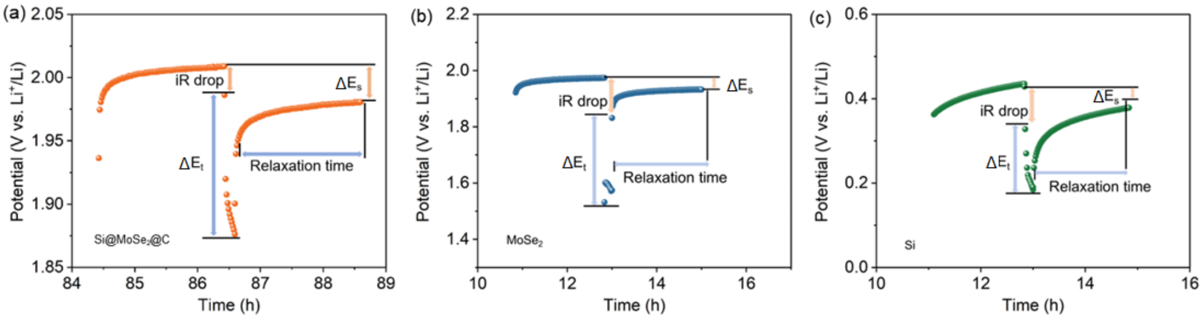

Fig. S27 The iR drop, ΔE_s, and ΔE_τ of (a) Si@$MoSe_2$@C, (b) $MoSe_2$, and (c) Si.

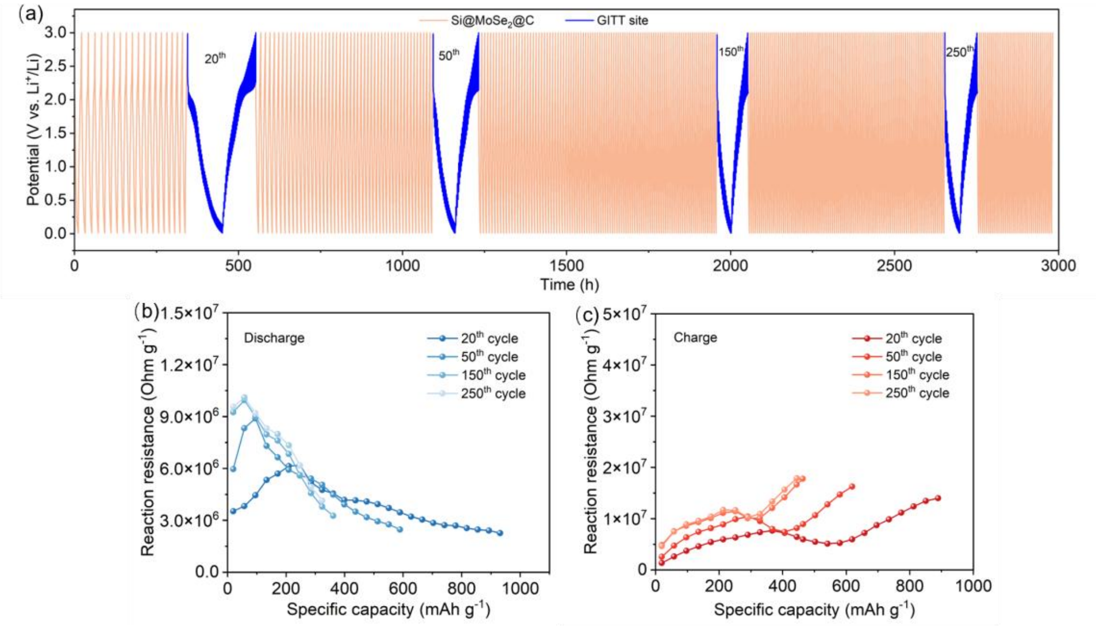

Fig. S28 (a) Voltage vs. time profile of the Si@$MoSe_2$@C anode during GITT measurement at 0.2 A g^{-1}. Corresponding *in-situ* reaction resistances during (b) discharge and (c) charge in GITT tests at different cycles.

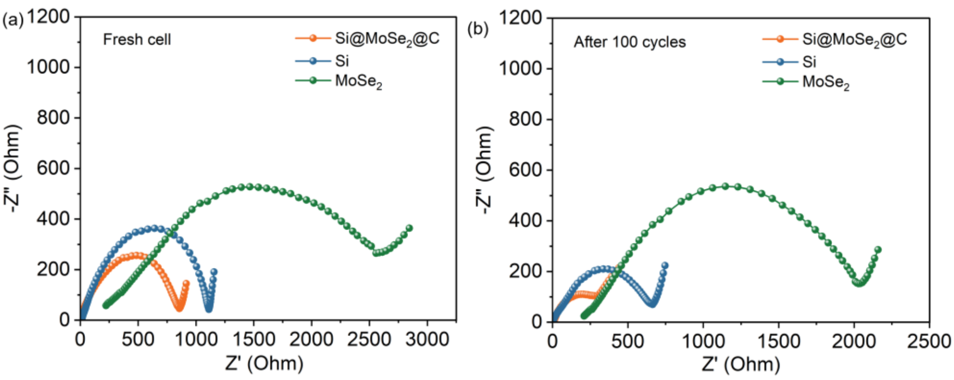

Fig. S29 (a) EIS spectra of fresh cells with different anodes. (b) EIS spectra for different anodes after 100 cycles at 0.2 A g^{-1}.

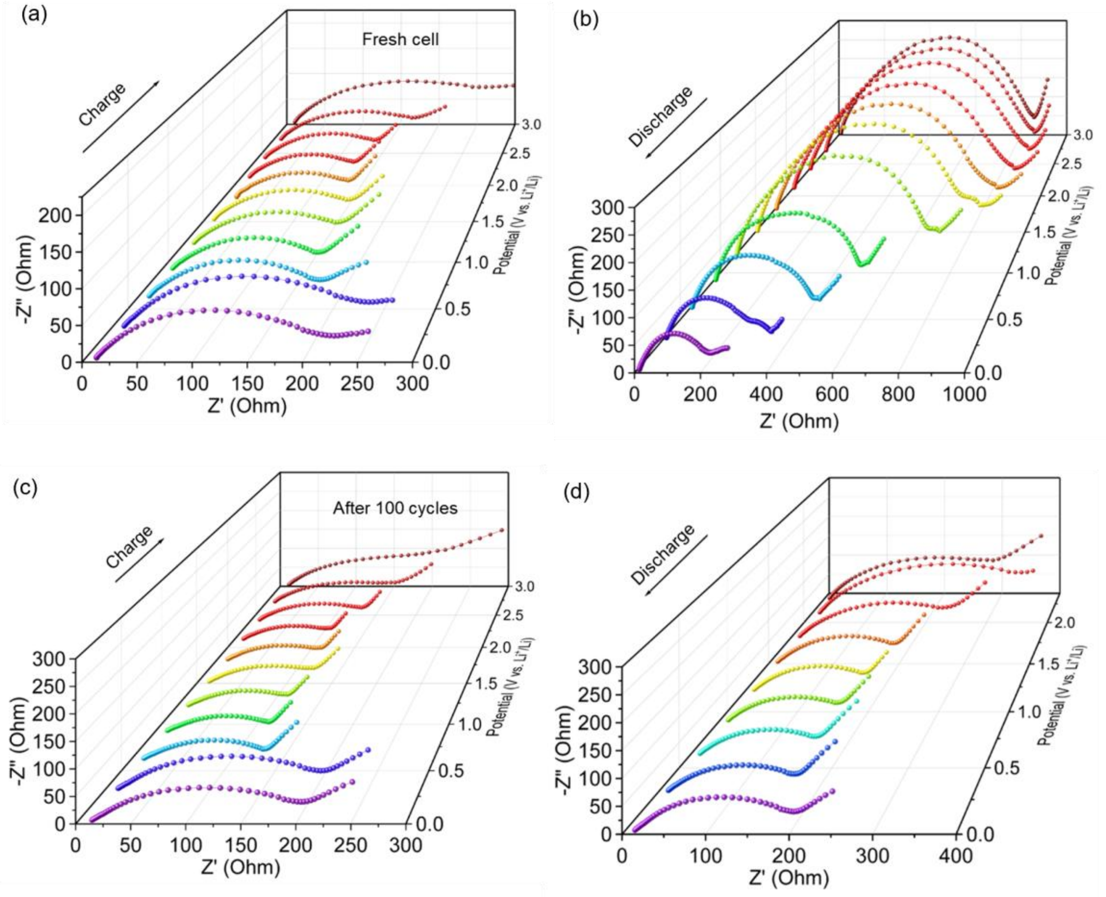

Fig. S30 (a,b) *In-situ* impedance spectra of Si@$MoSe_2$@C-based fresh cell at different discharge/charge voltage. (c,d) *In-situ* impedance spectra of the cell after 100 cycles at 0.2 A g^{-1} at different discharge/charge potentials.

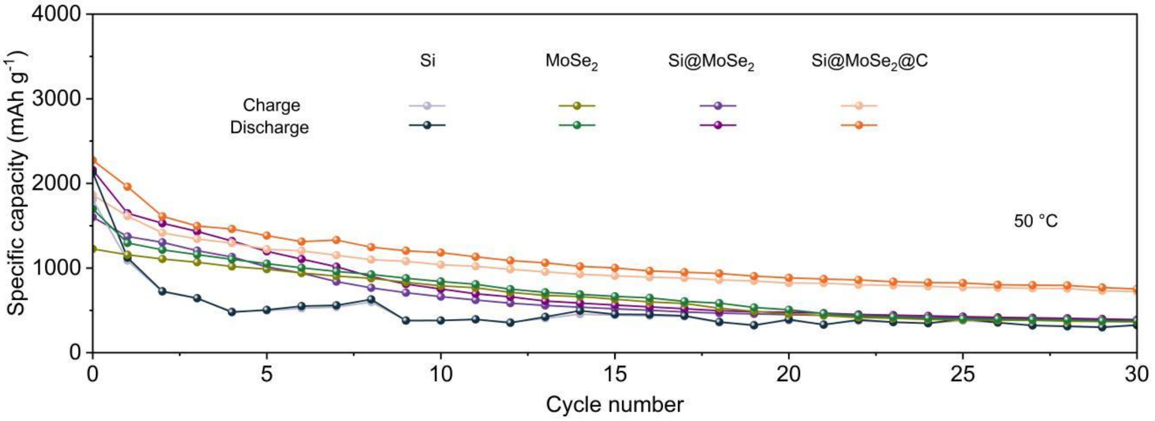

Fig. S31 Cycling performance of different anodes at 0.2 A g^{-1} at 50 °C.

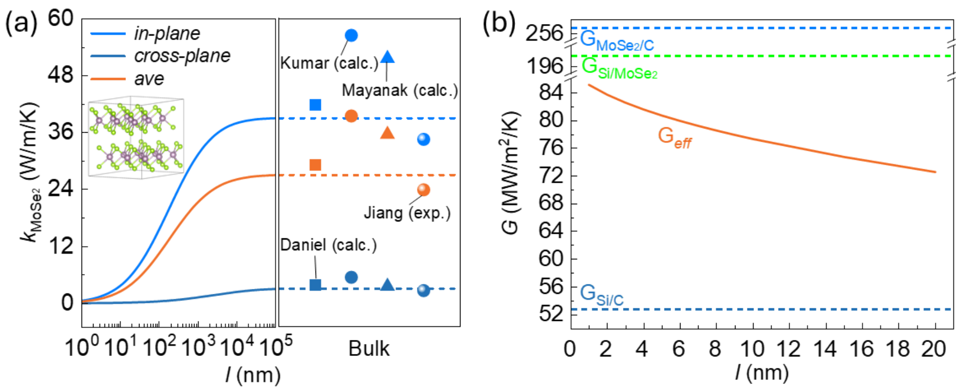

Fig. S32 (a) Thickness-dependent k_{MoSe_2}. Previously reported k_{MoSe_2} [16–19] are shown for comparison. (b) Thickness-dependent G_{eff} (solid line). $G_{Si/C}$, $G_{MoSe_2/C}$, and $G_{Si/MoSe_2}$ (dashed line) are shown for comparison.

Table S1. Comparison on electrochemical performance of some Li-ion battery anodes.

Anodes	Current density ($A\ g^{-1}$)	Capacity ($mAh\ g^{-1}$)	Cycle number	Ref.
$Si@MoSe_2@C$	1.0	782	400	This work
$C\text{-}SiO_x/C$	0.09	836	500	[34]
SiO_x@graphene/ graphite	0.37	400	50	[35]
$C\text{-}SiO_x@Si/rGO$	1.0	925	100	[36]
Porous rod-like Si-C	0.1	769	31	[37]
Si/carbon nanorod	0.1	600	100	[38]
Nanoporous Si/graphite	0.5	328	100	[39]
Si CNFs-200	0.1	536	100	[40]
Plate-like Si@C	0.1	402	100	[41]
$Si@void@TiO_2$	0.42	740	100	[42]

Table S2. Thermal properties (k and C) and thickness (t) of the materials used in 3ω method. For the PI, both in-plane (k_{in}) and out-of-plane (k_{out}) thermal conductivities were measured using 3ω method and verified by frequency-domain thermoreflectance (FDTR), while the C was taken from literature,[43] and the thickness was measured using a micrometer. The k and C of PMMA were obtained from literature,[44,45] and its thickness was measured using a surface profiler. For porous Si and porous $Si@MoSe_2@C$-based matrix composite, the ks were treated as fitting parameters in the model. Their specific heat capacities (c_p) and densities (ρ) were determined by differential scanning calorimetry (DSC) and a coupled mass and volume measurements, respectively. The Cs were then calculated as $C=c_p*\rho$. Their thicknesses were measured using a micrometer.

Materials	k (W m^{-1} K^{-1})	C (MJ m^{-3} K^{-1})	t (μm)
PI	k_{in} = 0.58<br>k_{out} = 0.38	1.72	300
PMMA	0.19	1.58	1.54
Si based matrix	Fitting parameter	1.06	300
$Si@MoSe_2@C$-based matrix	Fitting parameter	1.77	300

The specific parameters used for each layer in the multilayer model used for fitting are summarized in Table S2. By fitting the measured in- and out-of-phase signal of the $T_{2\omega}$ over a range of frequencies to the multilayer heat conduction model, the k of the sample could be accurately extracted.

References

[1] E. Yamasue, M. Susa, H. Fukuyama, K. Nagata, *J. Cryst. Growth* **2002**, *234*, 121−131.

[2] P. Jiang, X. Qian, X. Gu, R. Yang, *Adv. Mater.* **2017**, *29*, 1701068.

[3] C. Yuan, J. H. Li, L. Lindsay, D. Cherns, J. W. Pomeroy, S. Liu, J. H. Edgar, M. Kuball, *Commun. Phys.* **2019**, *2*, 43.

[4] M. Li, J. S. Kang, Y. Hu, *Rev. Sci. Instrum.* **2018**, *89*, 084901.

[5] A. J. Schmidt, X. Chen, G. Chen, *Rev. Sci. Instrum.* **2008**, *79*, 114902.

[6] V. Mishra, C. L. Hardin, J. E. Garay, C. Dames, *Rev. Sci. Instrum.* **2015**, *86*, 054902.

[7] S. Torquato, *Interdiscip. Appl. Math.* **2002**, *16*, 459−484.

[8] S. Bu, B. Chen, Z. Li, J. Jiang, D. Chen, *Nucl. Eng. Des.* **2021**, *376*, 111106.

[9] S. J. Rodrigues, N. V. Huget, E. Tsotsas, *Int. J. Heat. Mass Tran.* **2022**, *194*, 122994.

[10] K. M. Chung, J. Zeng, S. R. Adapa, T. S. Feng, M. V. Bagepalli, P. G. Loutzenhiser, K. J. Albrecht, C. K. Ho, R. K. Chen, *Sol. Energ. Mat. Sol. C.* **2021**, *230*, 111271.

[11] B. Graczykowski, A. E. Sachat, J. S. Reparaz, M. Sledzinska, M. R. Wagner, E. C. Angel, Y. Wu, S. Volz, Y. Wu, F. Alzina, C. M. S. Torres, *Nat. Commun.* **2017**, *8*, 415.

[12] D. G. Tsalikis, V. G. Mavrantzas, S. E. Pratsinis, *Phys. Fluids* **2023**, *35*, 097131.

[13] P. Reddy, K. Castelino, A. Majumdar, *Appl. Phys. Lett.* **2005**, *87*, 211908.

[14] Q. Song, G. Chen, *Phys. Rev. B* **2021**, *104*, 085310.

[15] Z. R. Han, X. L. Yang, W. Li, T. L. Feng, X. L. Ruan, *Comput. Phys. Commun.* **2022**, *270*, 108179.

[16] P. Jiang, X. Qian, X. Gu, R. Yang, *Adv. Mater.* **2017**, *29*, 1701068.

[17] M. K. Gupta, S. Kumar, R. Mittal, S. K. Mishra, S. Rols, O. Delaire, A. Thamizhavel, P. U. Sastry, S. L. Chaplot, *J. Mater. Chem. A* **2023**, *11*, 21864.

[18] S. Kumar, U. Schwingenschlögl, *Chem. Mater.* **2015**, *27*, 1278.

[19] D. O. Lindroth, P. Erhart, *Phys. Rev. B* **2016**, *94*, 115205.

[20] A. Savin, R. Nesper, S. Wengert, T. F. Fassler, *Angew. Chem. Int. Ed.* **1997**, *36*, 1808−1832.

[21] G. Kresse, J. Furthmüller, *Comp. Mater. Sci.* **1996**, *6*, 15.

[22] G. Kresse, J. Furthmüller, *Phys. Rev. B* **1996**, *54*, 11169.

[23] K. Momma, F. Izumi, *J. Appl. Cryst.* **2008**, *41*, 653.

[24] R. Dronskowski, P. E. Bloechl, *J. Phys. Chem.* **1993**, *97*, 8617.

[25] S. Maintz, V. L. Deringer, A. L. Tchougréeff, R. Dronskowski, *J. Comput. Chem.* **2016**, *37*, 1030.

[26] E. T. Swartz, R. O. Pohl, *Rev. Mod. Phys.* **1989**, *61*, 605.

[27] J. C. Duda, J. L. Smoyer, P. M. Norris, P. E. Hopkins, *Appl. Phys. Lett.* **2009**, *95.*

[28] Y. Xu, Y. Zhou, *Phys. Rev. B* **2024**, *110*, 115305.

[29] A. Togo, L. Chaput, T. Tadano, I. Tanaka, *J. Phys. Condens. Matter.* **2023**, *35*, 353001.

[30] J. M. Ziman, *Electrons and Phonons: The Theory of Transport Phenomena in Solids*, OUP Oxford, **2001**.

[31] G. Chen, C. L. Tien, X. Wu, J. S. Smith, *J. Heat Transfer* **1994**, *116*, 325.

[32] D. L. Nika, E. P. Pokatilov, A. S. Askerov, A. A. Balandin, *Phys. Rev. B* **2009**, *79*, 155413.

[33] T. Brezesinski, J. Wang, S. H. Tolbert, B. Dunn, *Nat. Mater.* **2010**, *9*, 146−151.

[34] G. Li, L. B. Huang, M. Y. Yan, J. Y. Li, K. C. Jiang, Y. X. Yin, S. Xin, Q. Xu, Y. G. Guo, *Nano Energy* **2020**, *74*, 104890.

[35] L. Lee, Wang, W. T. A. Ran, J. H. Lee, S. M. Hwang, Y. J. Kim, *Chem. Eng. J.* **2022**, *442*, 136166.

[36] T. Meng, B. Li, Q. S. Wang, J. N. Hao, B. B. Huang, F. L. Gu, H. M. Xu, P. Liu, Y. X. Tong, *ACS Nano* **2020**, *14*, 7066−7076.

[37] L. F. Guo, S. Y. Zhang, J. Xie, D. Zheng, Y. Jin, K. Y. Wang, D. G. Zhuang, W. Q. Zheng, X. B. Zhao, *Int. J. Miner. Metall. Mater.* **2020**, *27*, 515−525.

[38] X. R. Li, H. P. Su, C. Ma, Y. C. Cong, J. Wang, H. Z. Lin, Y. Z. Shang, H. L. Liu, *Mater. Lett.* **2022**, *324*, 132636.

[39] Y. B. Liu, X. Y. Liu, Y. L. Zhu, J. W. Wang, W. W. Ji, X. Z. Liu, *Energy Fuels* **2023**, *37*, 4624−4631.

[40] X. H. Li, X. X. Wang, J. J. Li, G. Liu, D. C. Jia, Z. L. Ma, L. Zhang, Z. Peng, X. Y. Zhu, *Electrochem. Commun.* **2022**, *137*, 107257.

[41] S. Y. Zhang, J. Xie, C. Wu, X. B. Zhao, *Int. J. Electrochem. Sci.* **2020**, *15*, 6582−6595.

[42] W. Luo, Y. X. Wang, L. J. Wang, W. Jiang, S. L. Chou, S. X. Dou, H. K. Liu, J. P. Yang, *ACS Nano* **2016**, *10*, 10524−10532.

[43] N. Chowdhury, J. C. Sun, D. G. Cahill, *ACS Appl. Polym. Mater.* **2025**, *7*, 1440−1447.

[44] M. J. Assael, K. D. Antoniadis, J. Wu, *Int. J. Thermophys.* **2008**, *29*, 1257−1266.

[45] S. Rudtsch, U. Hammerschmidt, *Int. J. Thermophys.* **2004**, *25*, 1475−1482.
